# Constitutive and activation-dependent phosphorylation of lymphocyte phosphatase-associated phosphoprotein (LPAP)

**DOI:** 10.1371/journal.pone.0182468

**Published:** 2017-08-21

**Authors:** Natalia A. Kruglova, Tatiana D. Meshkova, Arthur T. Kopylov, Dmitriy V. Mazurov, Alexander V. Filatov

**Affiliations:** 1 Department of Biology, Lomonosov Moscow State University, Moscow, Russia; 2 Institute of Biomedical Chemistry, Moscow, Russia; 3 NRC Institute of Immunology FMBA of Russia, Moscow, Russia; McGill University, CANADA

## Abstract

Lymphocyte phosphatase-associated phosphoprotein (LPAP) is a small transmembrane protein expressed exclusively in lymphocytes. LPAP is a component of a supramolecular complex composed of the phosphatase CD45, the co-receptor CD4, and the kinase Lck. In contrast to its immunologically important partners, the function of LPAP is unknown. We hypothesized that the biological role of LPAP may be determined by analyzing LPAP phosphorylation. In the present study, we identified LPAP phosphorylation sites by site-directed mutagenesis, phospho-specific antibodies, and protein immunoprecipitation coupled to mass spectrometry analysis. Our results confirmed previous reports that Ser-99, Ser-153, and Ser-163 are phosphorylated, as well as provided evidence for the phosphorylation of Ser-172. Using various SDS-PAGE techniques, we detected and quantified a set of LPAP phosphoforms that were assigned to a combination of particular phosphorylation events. The phosphorylation of LPAP appears to be a tightly regulated process. Our results support the model: following phorbol 12-myristate 13-acetate (PMA) or TCR/CD3 activation of T cells, LPAP is rapidly dephosphorylated at Ser-99 and Ser-172 and slowly phosphorylated at Ser-163. Ser-153 exhibited a high basal level of phosphorylation in both resting and activated cells. Therefore, we suggest that LPAP may function as a co-regulator of T-cell signaling.

## Introduction

Lymphocyte phosphatase-associated phosphoprotein (LPAP), encoded by the gene protein tyrosine phosphatase, receptor type C associated protein (*PTPRCAP*), is a 206 amino acid (aa) small transmembrane protein with an apparent molecular weight of 32 kDa [1]. LPAP is located predominantly on the plasma membrane as part of a supramolecular complex and is tightly bound to the phosphatase CD45 via its transmembrane domain [2]. Two segments of LPAP are identified as putative binding domains, including a WW domain [3] and an acidic domain required for interaction with the kinase Lck [4,5]. Another component of the supramolecular complex is the co-receptor CD4 [6]. Although LPAP is located at the cell membrane in proximity to several immunologically important molecules, its function remains unclear.

It was assumed that LPAP may serve as a substrate for CD45 [7] or, alternatively, may regulate CD45 activity [8]. These hypotheses initially ignited interest in LPAP, but this interest dissipated upon generation of *PTPRCAP* knock-out (KO) mice and the associated uncertain and contradictory results. The absence of *PTPRCAP* may have an effect on antigen-specific signaling [9], whereas other results found that *PTPRCAP* was dispensable for signaling [10,11]. Since these results were reported, new data related to LPAP have appeared only sporadically and in the context of large-scale transcriptomic [12] and proteomic studies [13,14]; very little LPAP-specific work has been conducted.

Because LPAP is a phosphoprotein with its phosphorylation status dependent upon cell activation, we hypothesize that analyzing LPAP phosphorylation may provide insight into its function. There are several identified LPAP phosphorylation sites [15–24], but these data were gleaned from bulk large-scale proteomic studies, and, unfortunately, do not specify stoichiometry or functional significance of particular phosphosites. A new set of anti-LPAP monoclonal antibodies, generated in our laboratory [25] and tested on the 10^th^ human leukocyte differentiation antigen (HLDA) [26], provide an additional instrument for LPAP investigation. Using these antibodies, we identified at least five different LPAP phosphoforms in lymphocytes [27].

Detection of protein phosphorylation is widely used to monitor signal transduction during cell activation. For instance, T-cell receptor (TCR) stimulation of Jurkat cells leads to changes in the phosphorylation status of nearly 700 unique sites associated with signaling, cytoskeleton polarization, alternative splicing, and other processes [28]. LPAP was identified as one such protein that phosphorylation status was dependent on cell activation [16]. Phorbol 12-myristate 13-acetate (PMA), the activator of protein kinase C (PKC) and Ras pathways, also alters LPAP phosphorylation [29].

Through determination of LPAP phosphorylation sites and the conditions under which their phosphorylation occurs, as well as identification of the kinases and phosphatases involved, the biological function of this protein can be understood. Here, we conducted a small-scale study focused on LPAP posttranslational modifications. We experimentally confirmed the sites of previously reported LPAP phosphorylation and identified a new site of phosphorylation. We assigned diverse combinations of phospho variants to particular LPAP proteoforms and determined their stoichiometry. Finally, we found that the phosphorylation status of LPAP changes dynamically upon cell activation with PMA.

## Materials and methods

### Ethics statement

This study design was approved by the Ethics Committee of NRC Institute of Immunology, Russia. Blood samples were obtained from healthy volunteers with written consent for the use of their samples. Thymocytes were obtained from children undergoing corrective cardiac surgery. All parents gave written informed consent for participation in the study.

Animal handling and experimental procedures were approved by the Ethics Committee Board for Animal Research of NRC Institute of Immunology FMBA of Russia. Blood were collected via retro-orbital puncture in mice under sodium pentobarbital anesthesia, and all efforts were made to minimize suffering.

### Cells and antibodies

Human cell lines CEM, Jurkat, and HEK293T were maintained in RPMI 1640 medium or Dulbecco’s Modified Eagle’s Medium, supplemented with 10% fetal bovine serum, 2 mM L-glutamine, and 24 μg/mL of Gentamicin (all Paneko, Moscow, Russia). PBMCs from healthy donors were isolated by centrifugation on Ficoll/Paque (GE Healthcare) density gradient. Thymocytes were obtained with informed parental consent from children undergoing corrective cardiac surgery. Cell activation was performed for 30 min at 37°C in complete RPMI 1640 medium with 10 ng/ml 4-phorbol 12-myristate 13-acetate (PMA, Sigma) or with 1 μg/ml anti-CD3 mAb (clone OKT3, eBioscience). Control cells were treated with DMSO carrier. Anti-LPAP mAbs CL3 and CL7 were produced in our laboratory [25] and their specificity were tested on the 10^th^ HLDA [26]. Anti-tubulin mAb (12G10) was purchased from The Developmental Studies Hybridoma Bank.

### Plasmid construction

The coding region of human wild-type LPAP gene was synthesized and subcloned into the lentiviral expression vector pUCHR IRES GFP. The point mutations were produced by overlap extension PCR with primers bearing mutations The mutations introduced were the following: S99A (TCC codon changed to GCC), T113A (ACA changed to GCA), Y115 (TAT changed to GCT), S153 (AGT changed to GCT), T155A (ACG changed to GCG), S163A (TCC changed to GCC), S168A (AGC changed to GCC), S172A (AGT changed to GCT), S153Stop (AGT changed to TAG), S163Stop (TCC changed to TAG), S172Stop (AGT changed to TAG), A184Stop (GCT changed to TAG). All mutations were verified by DNA sequencing.

### Generation of LPAP deficient CEM cells

A pair of gRNAs targeting LPAP gene was designed using web-resource www.genome-engineering.org [30]. Oligonucleotides CACCGCATCCCGAGCCCTAAGGTGC with AAACGCACCTTAGGGCTCGGGATGC, and CACCGCGCTGCCACCCGAGCCCAAG with AAACCTTGGGCTCGGGTGGCAGCGC were annealed, respectively, and cloned into gRNA expression plasmid pKS-gRNA-BB using BbsI restriction site. To minimize off-target effects, LPAP was knocked out using a double nicking technique [31]. 10^6^ CEM cells in 100 μL of buffer R (Invitrogen) were transfected with Neon electroporation system (Invitrogen) pulsing once at 1,230 V for 40 ms. Transfection mixture consisted of 3 μg of pcDNA3.3-Cas9 D10A plasmid (purchased from Addgene [32] and modified to nickase version) and 3 μg of each pKS-gRNA plasmids encoding LPAP specific gRNAs. Twenty-four hrs posttransfection, CEM cells were single-cell cloned into a 96-well plate. Three weeks later, single-cell clones were tested for LPAP expression in immunofluorescence. One clone with complete knockout of LPAP CEM was selected, named as CEM98, and used in all next experiments.

### Stable transfections

The CEM cells with the different LPAP mutants were generated by lentiviral transduction of CEM98 cells. For initiation of HIV-1 infection, HEK293T cells were cotransfected with 0.6 μg of HIV-1 packaging plasmid pCMVΔ8.2R (Addgene), 0.9 μg of pUCHR LPAP IRES GFP transfer vector, and 0.15 μg of pCMV-VSVG plasmid (Addgene) expressing Env G from vesicular stomatitis virus, using Lipofectamine 2000 (Invitrogene). The next day the culture medium was replaced, and cells were grown for another 24 h. Supernatant from the 6-cm dish with transfected HEK293T cells was harvested and clarified through a 0.45 μm pore size filter (Corning). Virus-like particles (VLPs) in the supernatant were concentrated by centrifugation at 100,000 g for 2.5 h and resuspended in 0.5 mL of fresh RPMI culture medium. Freshly prepared VLPs in a volume of 1 mL were added to 1 × 10^5^ CEM cells. Cells were grown for 4 days and sorted by GFP expression.

### Flow cytometric analysis and sorting

For intracellular staining, we used the protocol described in http://dx.doi.org/10.17504/protocols.io.h6zb9f6. Briefly, cells were washed with PBS and fixed with 1% paraformaldehyde (Sigma) in PBS for 5 min. Then, cells were permeabilized in PBS containing 0.1% saponin (Sigma) and 5% (w/v) nonfat milk for 30 min. Cells were stained using mAbs, conjugated to Alexa495. The stained cells were analyzed using a CytoFlex S flow cytometer (Beckman Coulter). The levels of fluorescence were measured and expressed as a mean fluorescence intensity. The cells treated with irrelevant mAb conjugated to Alexa495 served as a negative control. For sorting freshly prepared PBMC were stained with anti–CD4-PE and anti–CD8–FITC for 30 min. Thereafter, cells were washed once in PBS with 2% FCS, resuspended in PBS with 2% FCS, and sorted on a BD FACSAria II cytometer on CD4^+^ and CD8^+^ subpopulations.

### Immunoprecipitation

Labeling of cells with amino reactive fluorescent dyes Cy3 or Cy5 (BioDye, Moscow, Russia) was performed as described previously [27]. The protocol can be found at http://dx.doi.org/10.17504/protocols.io.h5db826. Briefly, 2 × 10^7^ cells were washed with PBS (pH 7.4) two times and resuspended in 1 ml of PBS. Then, 30 μL of cyanine succinimidyl ester stock solution in dimethyl sulfoxide (10 mg/mL) was added to the cells and incubated for 20 min. Unreacted dye was removed by washing cells with PBS two times. Cells were lysed in 1 mL of buffer containing 20 mM Tris-HCl (pH 8.0), 1% Triton X-100, 150 mM NaCl, 5 mM EDTA and 1 mM PMSF, 10 mM NaF and 1 mM Na_3_VO_4_ for 30 min at 4°C (all reagents were purchased from Sigma). Cell debris was pelleted at 20,000 g for 15 min at 4°C. Cell lysates were cleared overnight at 4°C by rotation with normal mouse IgG covalently linked to CNBr-Sepharose. Precipitations of precleared lysates with specific mAbs were carried out by using 30 μL 25% AffiGel Hz hydrazide agarose beads (Bio-Rad) coupled to anti-LPAP CL7 or CL3 mAb. Samples were precipitated under rotation at 4°C for 2 h. Then, the beads were washed four times in the lysis buffer. The proteins were eluted by heating the beads in the SDS sample buffer at 80°C for 5 min or in isoelectric focusing sample buffer containing 7 M urea, 2 M thiourea, 2% Triton X-100, 2% ampholytes, pH 3–10, and 100 mM DTT at 28°C for 2 h.

### Dephosphorylation

LPAP immune-purified with AffiGel-CL7 beads was transferred into the buffer containing 50 mM Tris-HCl (pH 7.6); 10 mM MgCl_2_; 100 mM NaCl; 1 mM DTT. Ten units of CIP (SibEnzyme) were added to 50 μL of this solution and incubated for 1 h at 37°C. Control reactions were performed at 37°C in the same buffer without CIP.

### Electrophoresis

Eluted samples were subjected to Laemmli SDS–PAGE, phosphate-affinity SDS–PAGE, or 2D electrophoresis. Laemmli SDS–PAGE was performed with 12% or 18% polyacrylamide gels under reducing conditions. Phos-tag SDS–PAGE was performed with 10% polyacrylamide gels containing 50 μM Phos-tag acrylamide (Wako, Osaka, Japan) and 100 μM ZnCl_2_. For 2D-DIGE samples labeled with Cy3 and Cy5 were mixed, loaded on 7 cm Immobiline DryStrip pH 4–7 (GE Healthcare) and separated in the first dimension using an isoelectric focusing system Ettan IPGphor 3 (GE Healthcare) as follows: 300 V for 30 min, 1000 V for 30 min (gradient), and 5000 V for 1.5 h (gradient), 5000 V for 30min. After isoelectric focusing, strips were equilibrated in reducing solution (50 mM Tris-HCl pH 6.8, 6 M urea, 30% glycerol, 2% SDS) containing 1% DTT for 15 min and then in the same buffer containing 5% iodoacetamide instead of DTT for 15 min. 2D-PAGE was performed using 18% resolving gels at 180 V for 3 h. Gels after 2D electrophoresis were visualized using an Amersham Imager 600RGB (GE Healthcare).

### Western blotting

Proteins from the gel were transferred to PVDF membrane with a semidry blotting system (Bio-Rad) for 30 min at 10 V. Membrane was blocked overnight at 4°C with 5% (w/v) nonfat milk in PBS containing 0.02% Tween 20, probed with anti-LPAP CL7 mAb conjugated to horseradish peroxidase. Blots were washed again, and immunoreactive bands were detected with Immobilon Western reagent (Millipore) on a Molecular Imager ChemiDoc XRS (Bio-Rad). In some experiments blots were probed with primary antibodies, washed with PBS-Tween and developed with horseradish peroxidase-conjugated secondary antibodies (Cell Signaling, Danvers, MA, USA).

### Mass Spectrometry

LPAP immune-purified from lysate of 5 × 10^8^ CEM cells was subjected to 10% SDS-PAGE. The position of the band containing unlabeled LPAP on the gel was identified by a parallel running of Cy3-labeled LPAP. Bands were manually excised and fixed in acetic acid. To remove SDS gel pieces were washed twice in 40% methanol with 10% glacial acetic acid and rinsed three times in deionized water. For trypsin digestion gel fragments were equilibrated with 50 mM triethyammonium bicarbonate buffer (pH 8.0). Trypsin (Promega) was added in a ratio of 1/50 (w/w) and samples were incubated at 42°C for 5 h. In case of pepsin digestion gel fragments were dried under vacuum, then rehydrated in 3% trifluoracetic acid with pH 1.0 or alternatively in 30 mM hydrochloric acid (pH 2.0–2.5). Pepsin (Sigma) was added in a ratio 1/30 and in a ratio 1/50 in 4 h after beginning of reaction. Digestion with pepsin was carried out at 37°C overnight. Peptides were extracted in three consequent change of 1% trifluoracetic acid and dried under vacuum. The pellet was reconstituted in 10–15 μL of 0.5% formic acid and transferred in glass inserts for LC-MS.

Liquid chromatography (LC) separation was performed on an Ultimate 300 RSLCnano system (Thermo Scientific) using an analytical Acclaim^®^ RSLC PepMap column (75 μm x 150 mm, 1.8 μm particle size, 100A pore size; Thermo Scientific) coupled with enrichment μ-precolumn C18 PepMap (300 μm x 5 mm, 5 μm particle size, 100A pore size; Thermo Scientific). Separation was carried out in eluting gradient of mobile phase A (water with 0.08% formic acid, 0.02% acetic acid; pH 2.7) and mobile phase B (acetonitrile with 0.08% formic acid and 0.02% acetic acid). The initial starting condition was 2% of B for 3.5 min following linear increasing to 35% of B for 37.5 min and hold in isocratic mode for the following 4 min.

The MS analysis was performed on Orbitrap Fusion (Thermo Scientific) mass spectrometer equipped with NSI ion source. The instrument was operated in the positive ionization mode. The capillary voltage was set at -2.1 kV, and the ion transfer tube temperature was 260°C. The electrodynamic ion funnel voltage (s-lens RF level) was adjusted to 75%. In the data-dependent analysis (ddMS2) the precursor ions were surveyed in a range of 420–1200 m/z at a resolution of R = 60K using quadrupole mass analyzer and Orbitrap detector type. The acquisition gain control (AGC) target was set to 4e5 ion, or maximum integration time of 85 ms. Dynamic exclusion after 3 consequent scans within 20 s (independent on single charge state per precursor) for 45 s and active apex triggered detection were applied during analysis. The precursor ions were selected for MS/MS using top-speed mode. The high-energy collision dissociation (HCD) was used as an activation type. Fragmentation was performed at 27% normalized HCD collision energy with stepped energy ramping within ±20%. Detection of the fragment ion was accomplished in Orbitrap detector type at a resolution of R = 15K at a normal scan speed using fixed first mass as 110 m/z. The AGC target was set to 5e4 ions, or maximum integration time of 60 ms. Full duty cycle time was 2.5 s.

After preliminary data-dependent survey and data processing, the further analysis was performed in targeted MS2 mode (t-MS2) at a resolution of R = 30K and AGC of 1e5–2.5e5 ions, or varied integration time of 125–200 ms. The normalized collision energy was set on 22–26% depending on the target precursor ion fragmentation efficiency inspected from the data-dependent analysis. Ions were isolated using quadrupole mass analyzer and the isolation window for all target ions was 3 m/z with offset of 0.5 m/z.

Spectra were processed and analyzed using MASCOT search engine version 2.2 for conventional proteins and peptides identification. Phosphorylated sites searching following further identification and annotation were accomplished in support of Xcalibur version 3.0.63 (Thermo Scientific) and Peptide Shaker version 1.16.0 (Compomics) supporting MS-GF and OMMSA search algorithms. Theoretical calculation of peptides decomposition was performed and validated using Spectrum Mill Workbench version B.04.01.141 (Agilent Technology).

### Generation of LPAP phospho-specific antibodies

Four phospho-peptides RAELGpSTDN (pS99), CAEEARDpSDTE (p153), CDLVLGpSPGPA (pS163), and ASAGGpSAEA (pS172) corresponding to sequences surrounding Ser-99, Ser-153, Ser-163, and Ser-172 in LPAP were synthesized (GenScript) with an additional cysteine residue at the N-terminus. Peptide sequences were confirmed by mass spectrometry. Peptides were coupled to BSA using bifunctional cross-linker succinimidyl 4-(N-maleimidomethyl)cyclohexane-1-carboxylate (Thermo Scientific). BALB/c mice were immunized with 70 μg of conjugate in Freund's complete adjuvant (Sigma) followed by two immunizations with 70 μg of conjugate in Freund's incomplete adjuvant. Seven days later sera were collected and tested for their reactivity with phosphorylated and dephosphorylated peptides in ELISA. Ten animals were used for each antigen. Animal handling and experimental procedures were approved by the Ethics Committee Board for Animal Research of NRC Institute of Immunology FMBA of Russia.

## Results

### LPAP is constitutively phosphorylated in both resting and proliferating lymphocytes

**Fig 1:**
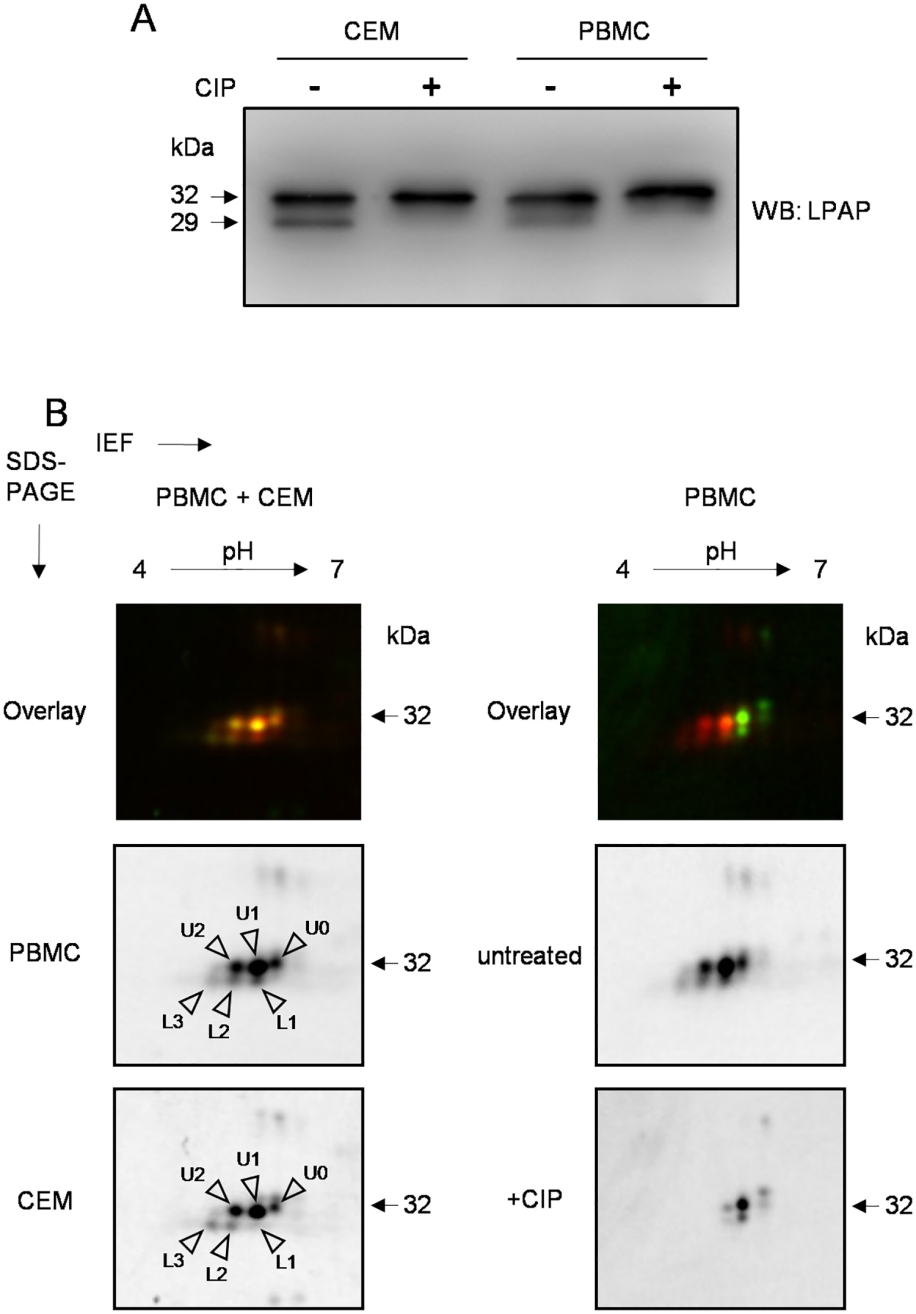
Comparison of LPAP phosphoforms from PBMCs and CEM cells. (A) LPAP immunoprecipitated from TX-100 lysates of PBMCs or CEM cells was dephosphorylated by CIP (+) or left untreated (-). Samples were subjected to 18% SDS-PAGE and Western blotting with anti-LPAP. (B) two-dimensional difference gel electrophoresis (2D-DIGE) analysis of LPAP immuno-purified from PBMCs (Cy5, red) and CEM cells (Cy3, green) (left column). 2D-DIGE of untreated (red) and dephosphorylated (green) LPAP from PBMCs (right column). Arrowheads indicate spots of LPAP phosphoforms. Spots are named U0 (phosphatase-resistant) and phosphatase-sensitive U1, U2 (upper train) and L1, L2, L3 (lower train) according to their relative mobility.

LPAP from Jurkat cells and other lymphoblastoid cell lines is phosphorylated [27,29], but whether this holds true for primary cells is unclear. To address this issue, freshly isolated peripheral blood mononuclear cells (PBMCs) were labeled with Cy3/Cy5, lysed, and LPAP was immunoprecipitated and analyzed by SDS-PAGE or difference gel electrophoresis (2D-DIGE). As shown in Fig 1A, LPAP from CEM cells, a T-lymphoblastoid cell line, and from PBMCs, migrated on 18% 1D-SDS-PAGE as two bands with apparent molecular masses of 28 and 32 kDa. Treatment of LPAP with calf intestinal phosphatase (CIP) resulted in disappearance of the lower band and an increase in the intensity of the upper band, indicating that the lower band corresponded to the phosphorylated protein. When resolved by 2D-SDS-PAGE, every band of LPAP split into at least three spots designated as U0, U1, and U2 for the upper train and L1, L2, and L3 for the lower train (Fig 1B). Phosphatase treatment collapsed the spots into one, overlaid with the most alkaline spot from the upper train in the untreated sample, suggesting that the spot designated as U0 represented unphosphorylated LPAP, while U1 and U2 were mono- and di-phosphorylated forms. Phosphorylation of an unknown residue resulted in the downward shift in electrophoretic mobility of LPAP and the formation of the lower train. Some high MW spots on 2D gels (Fig 1B) observed in fluorescent images were unspecific, because we could not detect them in Western blotting using anti-LPAP mAb.

Thus, LPAP was constitutively phosphorylated at a minimum of three sites and existed as five phosphoforms both in primary cells and in a proliferating lymphoblastoid cell line. Highly similar patterns of LPAP phosphorylation, in both primary cells and a CEM cell line, led us to conclude that CEM cells may serve as an adequate model for identification of LPAP phosphorylation sites in lymphocytes.

### Development of T-cell sublines expressing LPAP mutants

**Fig 2:**
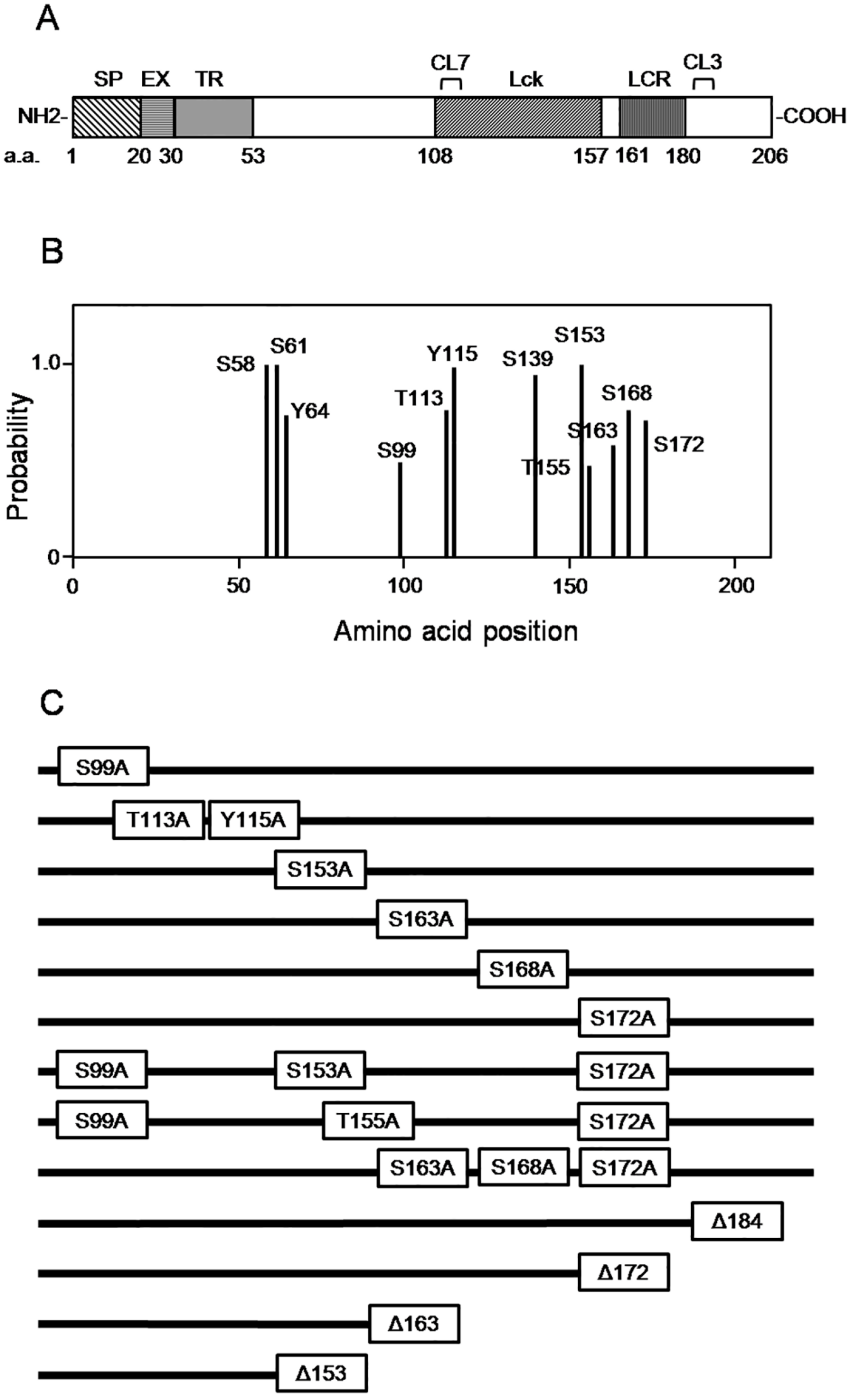
Schematic of LPAP structure with phosphorylation site prediction and representation of LPAP deletion and point mutants. (A) schematic representation of LPAP domains: SP, signal peptide; EX, extracellular; TR, transmembrane; Lck, Lck binding domain; LCR, low complexity region domain; a.a., amino acids; CL3, CL7, epitopes of anti-LPAP mAbs. Numbers indicate the position of amino acids. (B) phosphosites predicted by NetPhos 2.0. C, point and deletion LPAP mutants used in this work.

In order to identify potential phosphorylation sites, we used several web-based tools including NetPhos, [33] which predicted eight serine, two threonine, and two tyrosine residues (Fig 2A). Based upon these predictions, we generated a number of point and C-terminal deletion mutants of LPAP schematically shown in Fig 2B. In addition, we effectively inactivated the phosphorylation of Ser, Thr, and Tyr, by mutating them to Ala, mimicking a constitutive unphosphorylated state.

**Fig 3:**
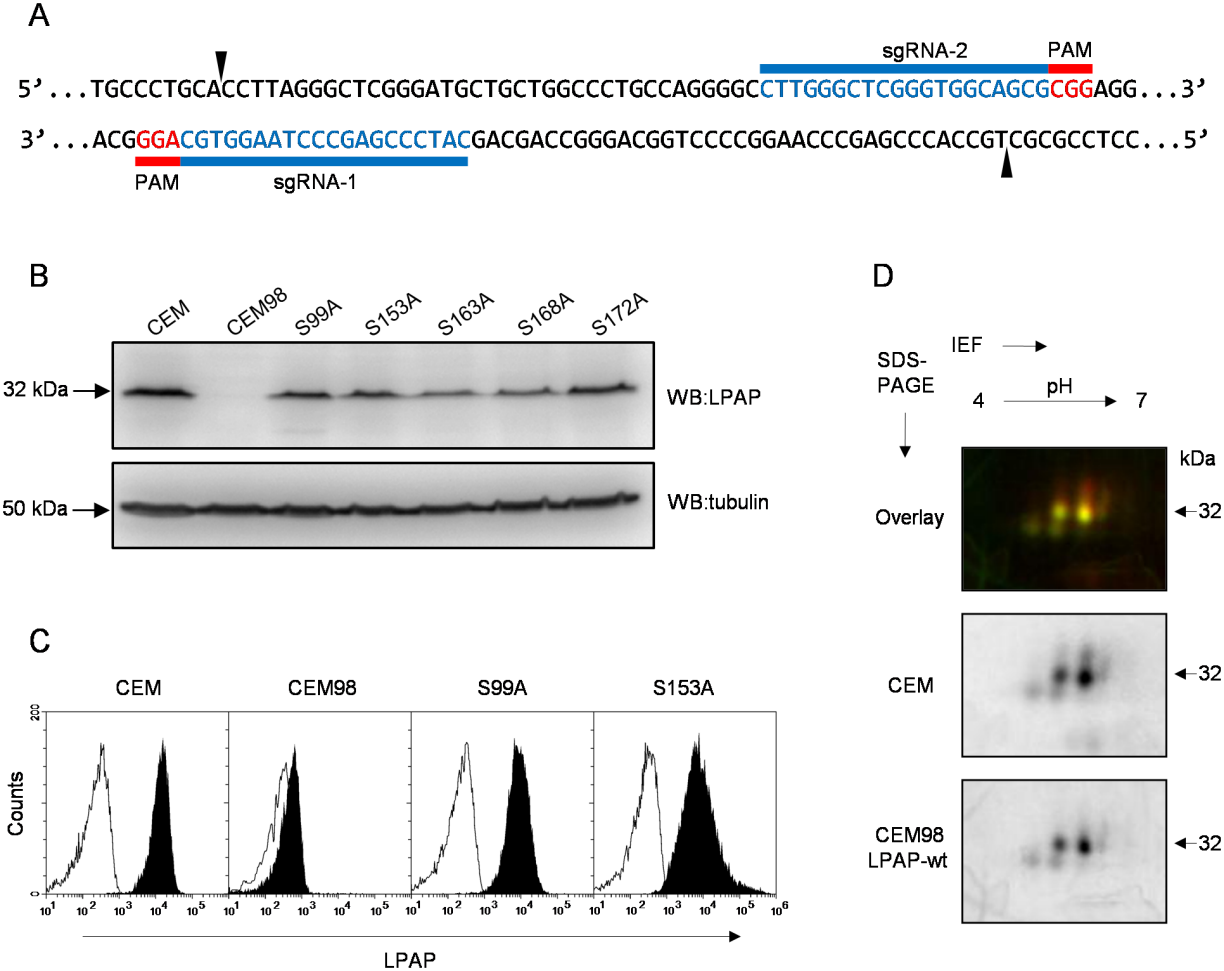
Generation and characterization of LPAP-deficient CEM cells stably transfected with mutant LPAP. (A) schematic of human LPAP gene targeting using Cas9 double-nicking strategy. Target regions of each sgRNA are labeled in blue, and PAM sequences are highlighted in red. (B) comparison of LPAP expression in parental CEM cells, LPAP-deficient clone CEM98, and LPAP-deficient cells stably transfected with LPAP mutants by Western blotting with anti-LPAP (upper panel), and by immunofluorescent staining of permeabilized cells (lower panel). Tubulin served as a loading control in Western blotting. Gray-filled histogram, isotype control in flow cytometry. C, 2D-DIGE comparison of LPAP proteoforms from parental CEM (green) and LPAP-deficient CEM98 cells stably transfected with wild-type LPAP (red).

LPAP mutant forms were expressed in a CEM T-cell line. As expression of endogenous LPAP in CEM cells may complicate analysis of mutant LPAP, we first generated LPAP-deficient CEM cells using clustered, regularly interspaced, short palindromic repeat (CRISPR)/Cas9 gene editing technology. Off-target effects were minimized using a double-nicking strategy [31] to KO LPAP, followed by single-cell cloning to isolate KO cells (Fig 3A). After clone verification by western blotting and immunofluorescence, we selected one clone, CEM98, for all subsequent experiments (Fig 3B). Using a lentiviral vector, we stably transduced the CEM98 cell line to produce sublines expressing LPAP mutants. LPAP expression in these cells varied from 50–100% relative to that detected in wild-type CEM cells (Fig 3B and 3C). When wild-type LPAP was re-expressed in LPAP-null CEM98 cells, 2D-electrophoresis of LPAP did not reveal any differences in patterns of LPAP migration when compared to LPAP isolated from CEM cells (Fig 3D). These data indicate that KO and transduction do not affect LPAP phosphorylation, and both exogenous and endogenous proteins were phosphorylated in a similar manner.

### Identification of LPAP phosphorylation sites by site-directed mutagenesis

**Fig 4:**
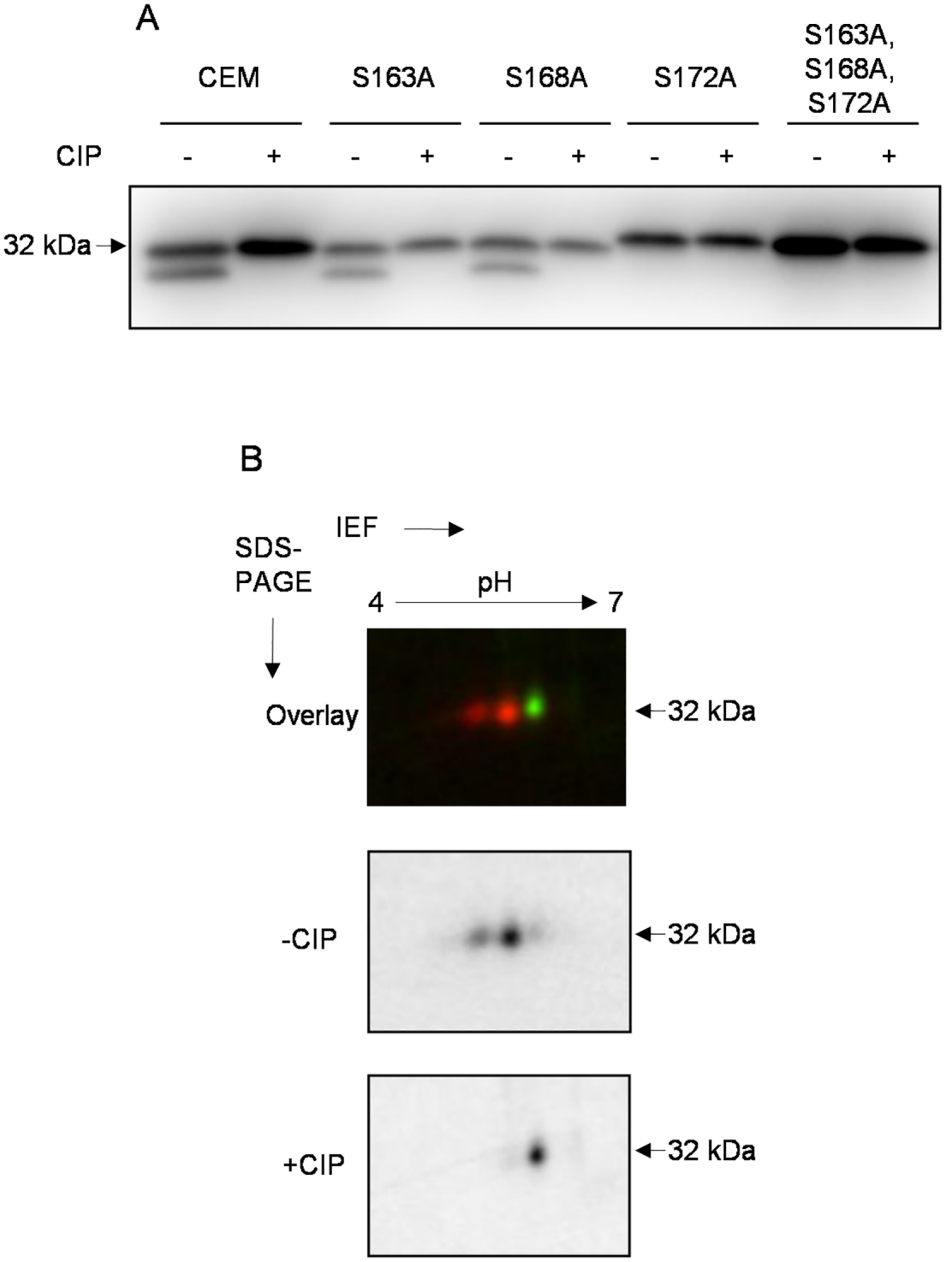
LPAP mutations that affect phosphorylation-dependent mobility shift. LPAP-deficient CEM cells stably transfected with LPAP were lysed in TX-100. Cell lysates were immunoprecipitated with anti-LPAP antibody. Samples were either treated (+) or untreated with the phosphatase CIP (-) as indicated and subjected to 18% SDS-PAGE or 2D-PAGE. (A) Western blot analysis of LPAP alanine point mutants. LPAP from parental CEM cells (lanes 1–2) was included for comparison. (B) 2D-DIGE analysis of S172A LPAP mutant. Cy3-labeled LPAP (green) was dephosporylated (indicated as +CIP), whereas Cy5-labeled LPAP (red) was left untreated (-CIP).

In addition to the major band at 32 kDa, LPAP had a downward-shifted satellite band at 29 kDa. This minor band disappeared following treatment with CIP, indicating that it corresponded to a phosphorylated form. To determine the phosphorylation site contributing to the downward shift, we analyzed different LPAP mutants. The point mutations, S163A and S168A, did not abolish the downward-shifted satellite band (Fig 4B, lanes 3–6). In contrast, the protein with the mutation at Ser-172 migrated as a single band (Fig 4B, lanes 7–10). The S172A mutant protein, resolved on a 2D-PAGE, had an unchanged spot pattern in the upper train but completely lacked spots in the lower train (Fig 4C). These data led us to conclude that Ser-172 was the phosphorylation site that affected LPAP electrophoretic mobility.

**Fig 5:**
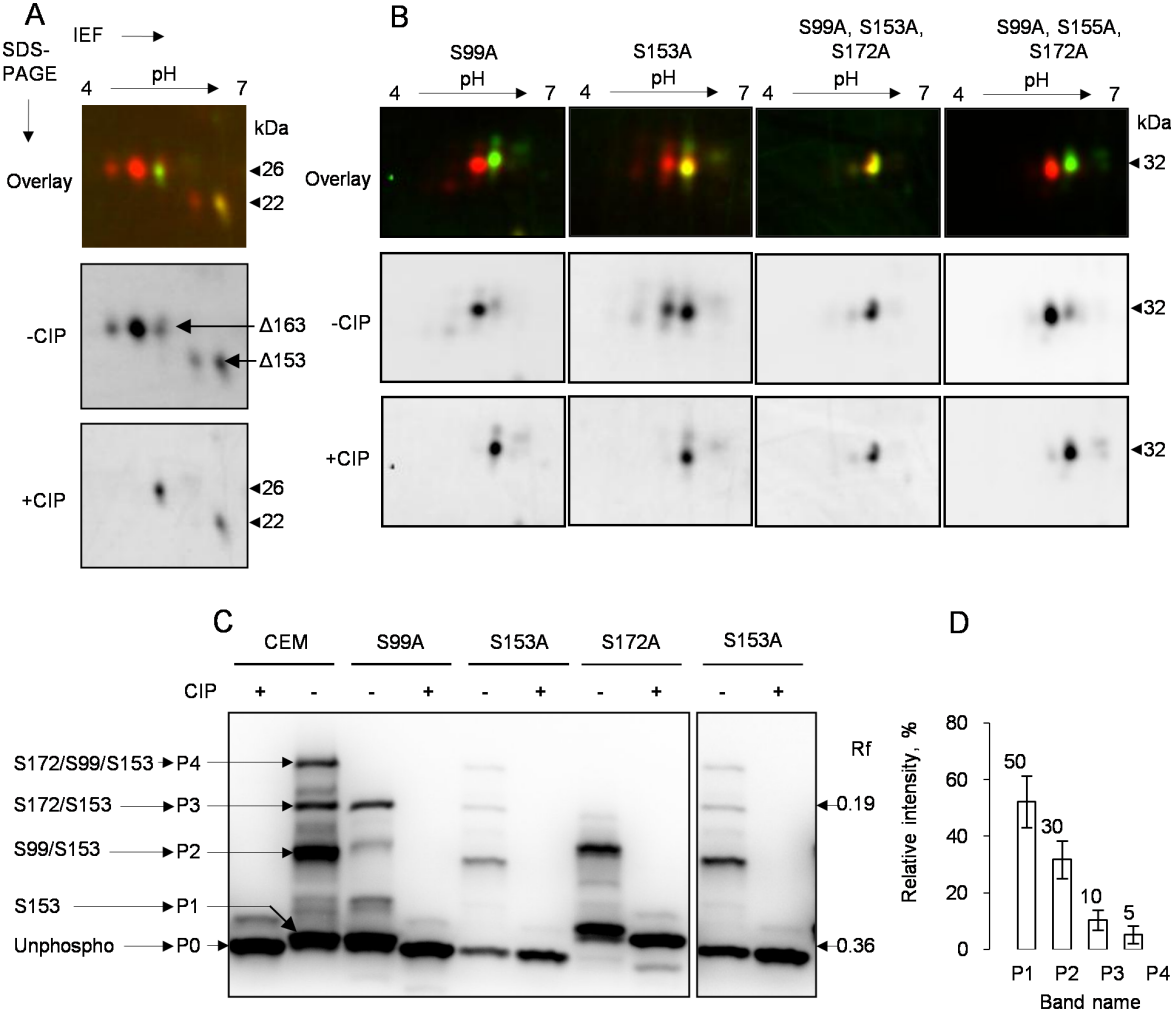
LPAP phosphorylation in resting CEM cells. LPAP-deficient CEM cells stably transfected with LPAP mutants were lysed in TX-100. Immunoprecipitated LPAP was either treated (+) or untreated (-) with the phosphatase CIP as indicated and subjected to 2D-DIGE or phosphate-affinity SDS-PAGE. For 2D-DIGE analysis LPAP was Cy3-labeled (green, CIP-treated) or Cy5-labeled (red, CIP-untreated). (A) 2D-DIGE analysis of LPAP Δ163–206 and Δ153–206 deletion mutants. Arrows indicate the positions of Δ163–206 and Δ153–206 deletion mutants, which were run on the same gel. (B) phosphorylation of S99A LPAP mutant (first column), S153A (second column), triple mutant S99A/S153A/S172A (third column), and triple mutant S99A/S155A/S172A (fourth column) detected by 2D-DIGE. (C) LPAP phosphorylation detected by Phos-tag SDS-PAGE. LPAP point mutants S99A (lanes 3–4), S153A (lanes 5–6, 9–10), S172A (lanes 7–8) or LPAP from parental CEM (lanes 1–2) were subjected to Phos-tag SDS-PAGE and blotted with anti-LPAP antibody. Lanes 9–10 were exposed for longer time to show minor bands (right panel). LPAP bands were named P0, P1, P2, P3, and P4 according to their relative mobility from fast to slow and assigned to the phosphorylation sites. The phosphorylation site(s) of each band were assigned as described on the left side: P0, unphosphorylated; P1, Ser-153; P2, Ser-99/Ser-153 and Ser-99; P3, Ser-172/Ser-153 and Ser-172; P4, Ser-99/Ser172/Ser-153 and Ser-99/Ser-172. (D) graph of band intensities ± s.d. (n = 5) in Phos-tag SDS-PAGE for resting CEM cells.

In order to define phosphorylation sites positioned more distally from the C-terminus, we analyzed the 2D-gel-mobility patterns of LPAP Δ163–206 and Δ153–206 deletion mutants. Significant differences between the molecular masses of these mutants allowed us to analyze them simultaneously on the same gel (Fig 5A). LPAP Δ163–206 had intact upper train but no lower train, while the shorter LPAP Δ153–206 retained only one phosphoform. Only two putative phosphorylation sites, Ser-153 and Ser-163, are located within the portion D152–S163 of LPAP. To differentiate between these residues, the S153A mutant was resolved on 2D-PAGE and, in comparison to the wild-type protein, produced only two forms in the upper train instead of three (Fig 5B, second column), while spots in the lower train were poorly detected. We tested another mutant, S99A, and found that both trains were reduced to one phosphorylated form (Fig 5A, first column).

We then analyzed two triple mutants by 2D-PAGE and found that S99A/S153A/S172A migrated as one spot (Fig 5B, third column), while S99A/S155A/S172A displayed two spots (Fig 5B, fourth column), one of which disappeared following CIP treatment. These results also indicate that Ser-153, but not Thr-155, is phosphorylated. Thus, the site-directed mutagenesis enabled us to conclude that Ser-99, Ser-153, and Ser-172 were the phosphorylation sites of LPAP.

Based upon our results, we assigned spots and bands to particular phosphoforms. The U0 spot on 2D-PAGE corresponds to unphosphorylated LPAP, U1 is a mono-phosphorylated form at either Ser-99 or Ser-153, and U2 represents a di-phosphorylated protein at both Ser-99 and Ser-153. All spots from the lower train were phosphorylated at Ser-172 and shifted into the acidic part of the gel. The L1 spot, which was very faint, represents the Ser-172 mono-phosphorylated form. The more intense spot, L2, corresponds to the mixture of di-phosphorylated forms pSer-99/pSer-172 with pSer-153/pSer-172. Finally, the most acidic spot, L3, comprises tri-phosphorylated LPAP pSer-99/pSer-153/pSer-172.

Unfortunately, 2D-PAGE has some limitations regarding the resolution of phosphoforms with the same number of phosphates. In order to overcome this obstacle, we used phosphate-affinity SDS-PAGE based on the interaction of phosphates with the Phos-tag molecules, which are co-polymerized in a gel and retard the mobility of phosphoproteins [34]. As different sites may have distinct surface exposure, and thus different binding to the Phos-tag reagent, proteoforms with the same number of phosphogroups can be separated. Using phosphate-affinity PAGE, we revealed phosphoforms that were impossible to detect by conventional 2D-PAGE. LPAP from wild-type CEM cells migrated on Phos-tag-PAGE as four major bands (Fig 5C, lane 2), P1, P2, P3, and P4, according to their relative mobility from fast to slow. In the phosphatase-treated sample, P2, P3, and P4 collapsed into the P0 band that was shifted slightly downwards relative to P1 (Fig 5C, lane 1). The S172A mutant did not have bands P3 and P4 (Fig 5C, lane 7), whereas the S99A mutant lacked P4, but the P2 band was present with reduced intensity (Fig 5C, lane 3). The S153A mutant had P0 instead of P1. Therefore, we assumed that phosphorylation at Ser-153 leads to the smallest mobility shift in Phos-tag-PAGE, and P1 corresponds to the mono-phosphorylated form at Ser-153. Ser-99 phosphorylation leads to a moderate mobility shift resulting in formation of P2, including mono-phosphorylated pSer-99 as well as di-phosphorylated pSer-99/pSer-153. Following this logic, Ser-172 phosphorylation produces the greatest shift and gives rise to P3, which contains mono-phosphorylated pSer-172 and di-phosphorylated pS172/pSer-153. Finally, the highest band, P4, represents the tri-phosphorylated form at Ser-99, Ser-153, and Ser-172.

The intensity ratio of the bands P2, P3, and P4 may be used to assess the stoichiometry of the phosphoforms bearing pSer-99, pSer-172, and pSer-99/pSer-172 sites (Fig 5D). The intensity of the first band represents the quantity of the unphosphorylated and pS153 mono-phosphorylated forms. Taking into account our previous results [27], demonstrating that the level of unphosphorylated LPAP was approximately 15%, we calculated the proportion of the pS153 mono-phosphorylated form to be approximately 40%.

Thus, our results obtained by two different electrophoretic methods are in agreement. Moreover, the combination of these methods enabled us to determine that the Ser-153 site is phosphorylated at a very high stoichiometry in unstimulated cells.

### Cell activation leads to changes in LPAP phosphorylation status

**Fig 6:**
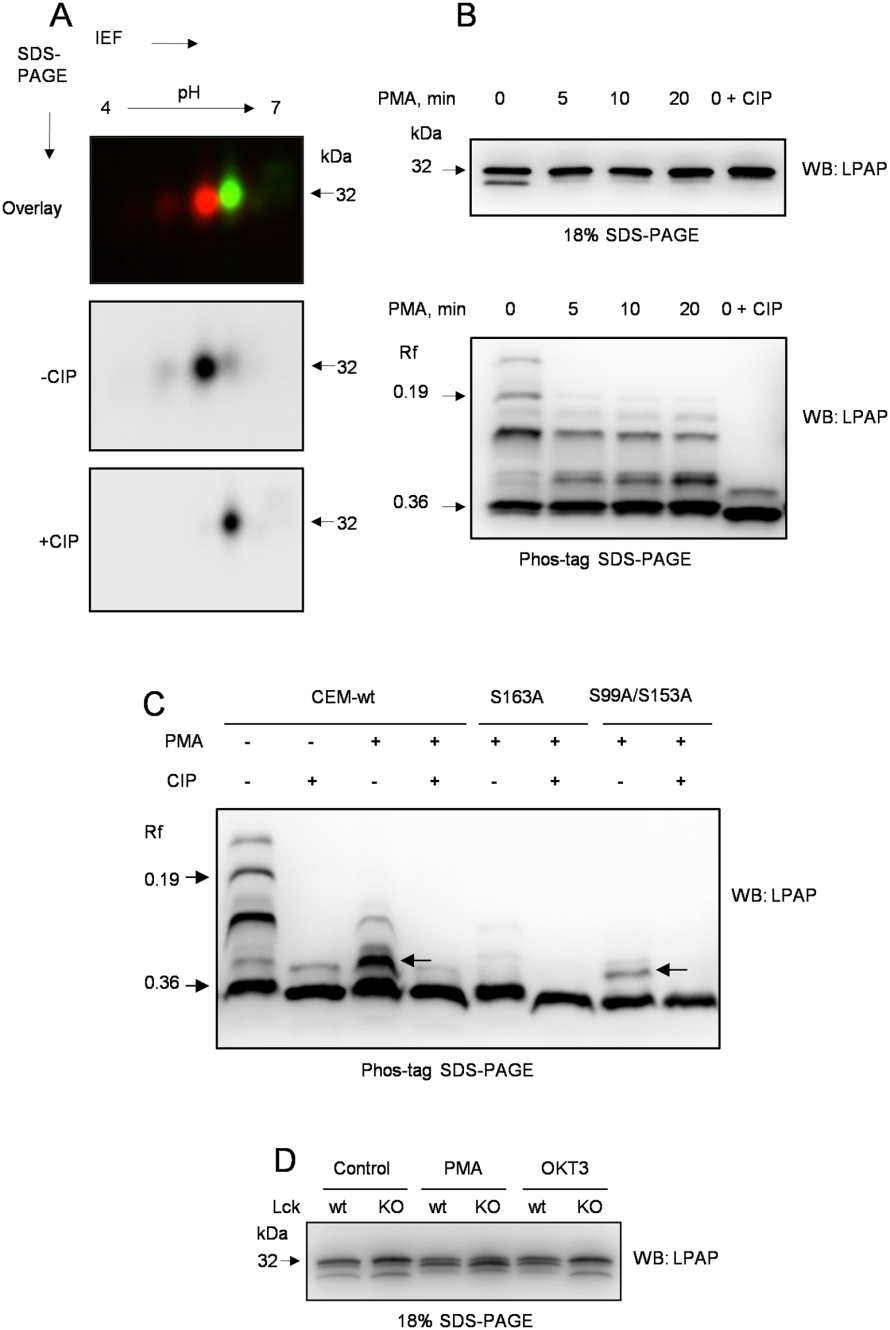
LPAP phosphorylation in activated CEM and Jurkat cells. (A) CEM cells were activated with PMA (10 ng/ml) for 30 min, lysed and immunoprecipitated with anti-LPAP antibody. Samples of phosphatase-treated (+CIP) Cy3-labeled (green) and untreated (-CIP) Cy5-labeled (red) were mixed, run on 2D-PAGE and visualized with a fluorescent gel scanner. (B) CEM cells were incubated for different times at 37°C with 10 ng/ml PMA and lysed. Immunoprecipitated protein was resolved on Phos-tag SDS-PAGE (upper panel) or conventional 18% SDS-PAGE (lower panel) and blotted with anti-LPAP antibody. Samples in lane 5 were not PMA-activated but treated with phosphatase (indicated as 0 + CIP). (C) phosphorylation of LPAP from PMA-activated parental CEM (lanes 3–4) or cells stably transfected with mutant S163A (lanes 5–6) or double mutant S99A/S153A (lanes 7–8) detected by phosphate-affinity SDS-PAGE. LPAP from resting parental CEM cells is included as a control (lanes 1–2). Arrows show activation-dependent bands in CEM-wt and S99A/S153A mutant, but not in S163A mutant. (D) Wild type Jurkat cells (wt) or Lck-deficient JCaM 1.6 cells (KO) were activated with PMA (10 ng/ml) for 30 min or OKT3 (1 μg/ml) mAb for 15 min, lysed and immunoprecipitated with anti-LPAP antibody. Immunopurified protein was resolved on 18% SDS-PAGE and blotted with anti-LPAP antibody.

The next aim of our study was to understand the functional significance of the identified phosphorylation sites and, in particular, to determine whether LPAP phosphorylation depends on external stimuli or cell activation status. Cell stimulation with PMA, a PKC activator, changes the phosphorylation status of LPAP [27,29]. Similarly, we observed a significantly altered pattern of LPAP phosphorylation after PMA treatment of CEM cells. The most prominent modification was the disappearance of the entire lower train on 2D-PAGE (Fig 6A) as well as of the lower band on 1D-SDS-PAGE (Fig 6B, upper panel). At the same time, the upper train was reduced to two spots, mono-phosphorylated and unphosphorylated. The kinetics of LPAP dephosphorylation was relatively quick, such that 5 min following PMA addition, the lower 29 kDa band disappeared on 18% 1D-PAGE, indicating that Ser-172 was dephosphorylated. In order to obtain more detailed information regarding the kinetics of LPAP phosphorylation, we performed Phos-tag-PAGE (Fig 6B, lower panel) and found that at 5 min of stimulation, the two upper bands, ascribed to Ser-172 phosphorylation, disappeared. Concurrently, the intensity of the band containing pSer-99 substantially decreased, and a new band appeared and increased in intensity until 20 min, after which it was maintained at the same level up to the end of the experiment. This new band migrated more slowly than the band with pSer-153 and was abrogated by phosphatase treatment. Thus, PMA stimulation led to phosphorylation of a new site on the LPAP molecule, and to identify this site, we tested additional mutants; however, the only mutation that abolished the PMA-triggered phosphoform was Ser-163A (Fig 6C). Noticeably, PMA activation resulted in a subtle downward shift of the mono-phosphorylated phosphoform on 2D-PAGE, which may be indicative of Ser-163 phosphorylation. Based upon these data, we conclude that after PMA stimulation, LPAP undergoes complete dephosphorylation at Ser-172, partial dephosphorylation at Ser-99, and phosphorylation at Ser-163. In contrast to PMA-dependent sites, the level of LPAP phosphorylation at Ser-153 remains very high in both resting and activated cells.

To investigate whether LPAP phosphorylation is dependent on TCR signaling, we performed CD3 stimulation of Jurkat T cells. We found that CD3 stimulation resulted in the loss of the band at 29 kDa and the appearance of the band at 30 kDa (Fig 6D). Thus, TCR-induced stimulation led to simultaneous dephosphorylation at Ser-172 and phosphorylation at Ser-163 residues. To confirm that LPAP phosphorylation changes were indeed caused by TCR signaling, we compared LPAP phosphorylation in wild-type Jurkat T cells and subline JCaM 1.6 (Lck-deficient Jurkat). These cells demonstrated similar type of phosphorylation/dephosphorylation after PMA-induced activation. Contrary to wild-type Jurkat cells, Lck deficient cells were unable to initiate effective TCR dependent LPAP phosphorylation (Fig 6D).

### Identification of LPAP phosphorylation sites by tandem mass spectroscopy (MS)

Next, we conducted MS analysis of LPAP to confirm identified phosphorylation sites in the native protein without any preliminary suggestions and mutations in the protein sequence. In spite of its small size, LPAP is a challenging protein to analyze for posttranslational modifications by MS. LPAP has no Lys residues and can be cut only at rare Arg residues, resulting in relatively large peptides, particularly in the area of interest. The phosphorylation site Ser-99 was easily identified because it was located on a MS-friendly tryptic peptide 95-AELGpSTDNDLER-106 (Table 1). However, the majority of the predicted phosphorylation sites are found on two very large tryptic fragments with molecular masses of 4287 and 4145 Da. The most poorly detected peptide was that with pS153 due to its sizable length and slow chromatographic mobility. This peptide was eluted in a far hydrophobic segment of the applied gradient. The reasonable signal of this peptide, with high accuracy suitable for the purpose of spectrum match sequencing, was obtained for an ion with a z = 4+ charge state. We did not observe any other modifications on the tryptic peptides and used pepsin digestion under strong acidic conditions in subsequent experiments. In this case, the peptide (aa 160–177) was the most interesting, because it was expected to bear a new phosphorylation site. Modification of the S172 residue was confirmed by a ladder of b-type fragment ions carrying neutral losses of 98 Da, which is a feature of a phospho-moiety. The y-type ions were poorly represented, because non-tryptic peptides are poorly ionized.

**Table 1:** Identification of LPAP phosphorylation sites by tandem mass spectrometry.

Peptide	Digestion	Phosphosite	Charge (z)	Observed m/z	Calculated m/z	Accuracy (ppm)	Retention time (min)	X corrected (for peptide)
95-AELG**pS**TDNDLER-106	trypsin	Ser-99	2+	700.2901	700.2905	-0.60	19.43	8.37
147-AEEARD**pS**DTEGDLVLGSPGPASAGGSAEALLSDLHAFAGSAAWDDSAR-194	trypsin	Ser-153	4+	1195.7877	1195.7878	+0.22	44.79	5.43
160- VLGSPGPASAGG**pS**AEALL-177	pepsin	Ser-172	2+	817.3951	817.3953	-0.13	31.29	6.17
160-VLG**pS**PGPASAGGSAEAL-176	pepsin	Ser-163	2+	760.8533	760.8538	+0.65	37.05	9.16
			3+	507.5713	507.5716	+0.59		7.71

In order to identify sites phosphorylated upon cell stimulation, we used samples of LPAP immuno-purified from PMA-stimulated CEM cells. The samples were subjected to pepsin digestion under pH~3.0, when the enzyme tends to have less cleavage specificity. Under these conditions, we detected a phosphopeptide, 160-VLGpSPGPASAGGSAEAL-176, as well as its intact counterpart. The peptide was successfully registered as an ion with double-charge and triple-charge states (Table 1) with high-mass accuracy for both states. There were two Ser residues within the peptide, but this ambiguity was resolved by rigorous investigation of fragmentation spectra. The location of the phosphogroup at S163 was elucidated by a series of b-ions starting from the b4 fragment, because some (such as b4, b5, b7, b9, b11) carried the phosphogroup, as well as ions with neutral losses of a water molecule and a phosphomoiety. However, because pS163 was located close to the N-termini, the vast majority of y-series ions were detected intact and only a small portion (y14, y15, and y16) could be fixed as phosphogroup carriers. Thus, the data on LPAP phosphorylation sites, derived by site-directed mutagenesis, were independently confirmed by MS.

### Verification of LPAP phosphorylation sites by phospho-specific antibodies

Phosphorylation sites were further validated through generation of phospho-specific antibodies in mice. Ten mice were immunized with one of four phosphopeptides that included regions of LPAP around Ser-99, Ser-153, Ser-163, and Ser-172. Mouse sera, with the highest specificity in binding phosphorylated peptide versus unphosphorylated analog by ELISA, were selected. Each phospho-specific antibody was named according to the phosphorylated amino acid residue of the immunizing phospho-peptide: pS99, pS153, p163, and pS172.

**Fig 7:**
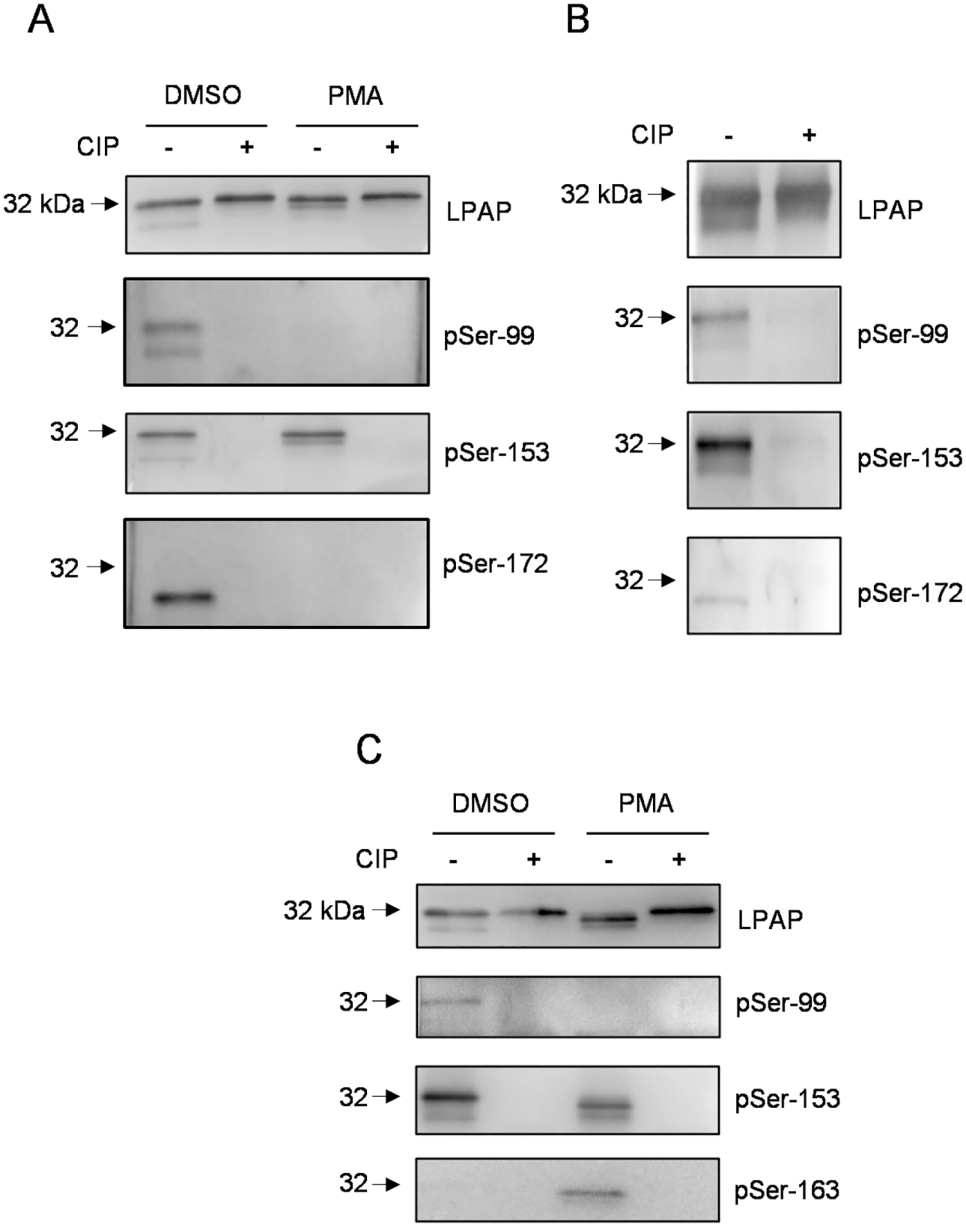
LPAP phosphoforms detection with phospho-site-specific antibodies. CEM cells or PBMCs were activated with PMA (10 ng/ml) for 30 min or left untreated. LPAP immunoprecipitated from CEM cells (A), thymocytes (B) or PBMCs (C) was analyzed by 18% SDS-PAGE followed by Western blotting using monoclonal anti-LPAP (top) or phospho-specific mouse antisera pSer-99, pSer-153, pSer-163, and pSer-172. Samples were dephosphorylated with CIP or left untreated (indicated as + or −).

Antisera pS99, pS153, and pS172 detected intact LPAP from resting CEM cells and demonstrated no staining with CIP-dephosphorylated LPAP upon western blotting (Fig 7A, lanes 1–2). Antisera pS99 and pS153 detected both LPAP bands at 32 and 29 kDa. By contrast, the pS172 antiserum stained a single band at 29 kDa. These data are in agreement with our previous results showing that the pSer-99 and pSer-153 modifications are present in both the 29 and 32 kDa bands, whereas phosphorylated Ser-172 was contained only in the 29 kDa band. PMA activation of CEM cells abolished the binding of pS99 and pS172 phospho-specific antibodies to LPAP (Fig 7A, lanes 3–4), whereas pS153 antiserum still reacted to the protein. Immunoblotting with phosphorylation-independent CL7 antibody served as a control for LPAP loading (Fig 7A, upper panel).

Similarly, phospho-specific antibodies pS99 and pS153 reacted with LPAP from thymocytes (Fig 7B) and resting or PMA-activated PBMCs (Fig 7C). LPAP, isolated from PBMCs, was not detected with p172 antiserum, whereas LPAP isolated from thymocytes was clearly visualized with p172 antiserum (Fig 7B, lower panel). p163 antisera stained single band only from PMA-activated CEM cells or PBMCs, but not from resting cells (Fig 7A and 7C). Thus, phospho-specific sera not only verified the phosphorylation sites identified by site-directed mutagenesis and MS, but were also suitable for detection and analysis of LPAP phosphorylation in primary cells.

### Activation-dependent LPAP phosphorylation in PMA-stimulated PBMCs

**Fig 8:**
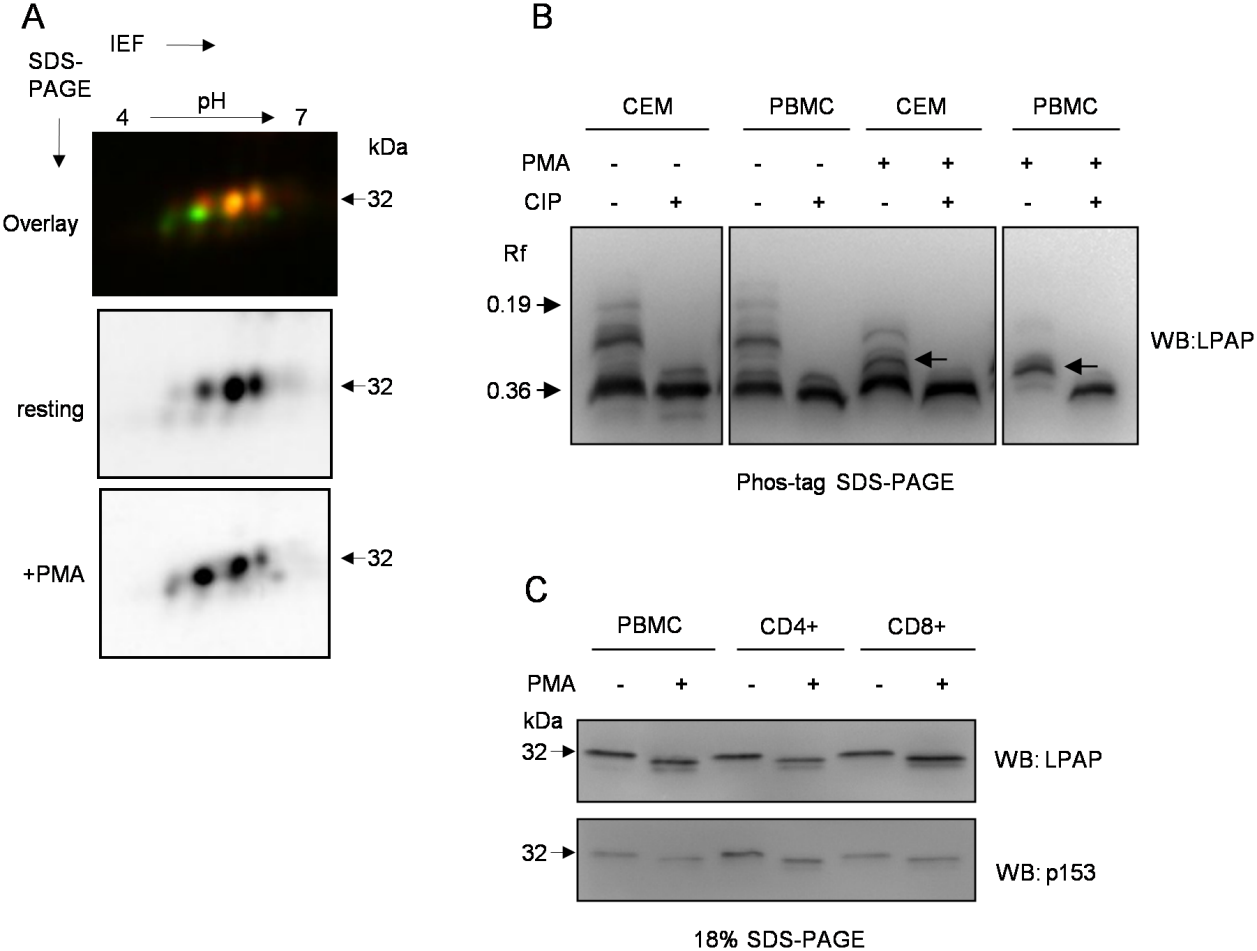
LPAP phosphorylation in PMA-activated PBMCs. PBMCs were activated with PMA (10 ng/ml) for 30 min or left untreated, lysed and immunoprecipitated with anti-LPAP antibody. (A) samples from PMA-activated Cy3-labeled cells and resting Cy5-labeled cells were mixed, run on 2D-PAGE and visualized with a fluorescent gel scanner. (B) samples from resting or PMA-activated PBMCs were either treated (+) or untreated with the phosphatase CIP (-) and analyzed by Phos-tag SDS-PAGE followed by Western blotting using monoclonal anti-LPAP. LPAP from CEM cells is presented for comparison. Arrows indicate the band, which appears after PMA activation. (C) PBMC and purified CD4^+^ or CD8^+^ cells were activated with PMA (10 ng/ml) for 30 min or left untreated, lysed, resolved on 18% SDS-PAGE and blotted with anti-LPAP or p153 antibodies.

In order to determine the phosphorylated sites of LPAP in activated PBMCs, we examined the effects of PMA treatment on the LPAP 2D-SDS-PAGE pattern. Similar to LPAP obtained from the CEM cells, LPAP from PMA-stimulated PBMCs lost the lower train of spots (Fig 8A), which can be interpreted as the dephosphorylation of Ser-172. The upper train maintained mono- and di-phosphorylated spots. Based upon our results with phospho-specific antibodies, Ser-153 was permanently phosphorylated, while Ser-99 was dephosphorylated in PMA-activated PBMCs. One explanation for the presence of the di-phosphorylated form, in the 2D-PAGE pattern of LPAP from PBMCs, is the existence of another phosphorylation site different from pSer-99 and pSer-172. As shown by 18% 1D-SDS-PAGE (Fig 7C, lane 3), LPAP from PMA-activated PBMCs formed a new band that was shifted slightly downward and disappeared after treatment with CIP (Fig 7C, lane 4). These results support our hypothesis that LPAP may have a fourth site of phosphorylation.

To further characterize the nature of activation-dependent phosphorylation, we separated LPAP from activated and resting cells by phosphate-affinity SDS-PAGE. After PMA stimulation of PBMCs, the bands assigned to pSer-172 bearing phosphoforms disappeared, the intensity of the band related to pSer-99 decreased, and a new band became visible (Fig 8B, lane 7). Electrophoretic mobility of this new band was very close to that of the LPAP band from PMA-activated CEM cells, which we ascribed to the pSer-163 phosphoform.

Next, we compared LPAP phosphorylation patterns in PBMC and in CD4^+^ and CD8^+^ subsets. PBMC and purified CD4^+^ and CD8^+^ cells demonstrated similar levels of Ser-153 phosphorylation both in resting and stimulated conditions (Fig 8C). Under PMA treatment, the band at 30 kDa representing Ser-163 phosphorylation was detected in all three cell populations. The band corresponded to Ser-172 phosphorylation was clearly revealed in PBMC but was not visible in CD4^+^ and CD8^+^ cells.

In conclusion, phosphorylation of LPAP in PBMCs, CD4^+^ and CD8^+^ subsets have a pattern very similar to that observed in CEM cells both in untreated and under PMA-stimulated conditions. Specifically, Ser-99 and Ser-172 become dephosphorylated, whereas Ser-163 becomes phosphorylated.

## Discussion

In accordance with its name, LPAP is a highly phosphorylated protein. Large-scale proteomics analyses have identified a number of LPAP phosphorylation sites, including Tyr-64, Ser-85 [20], Ser-99 [15–19,22–24], Thr-113 [24], Tyr-115 [24], Ser-153 [23], Thr-155 [19,24], and Ser-163 [13]. Despite a large body of data, none of these sites have been validated using site-specific methods. It is worth to note, that only half of the protein modification sites, determined by global MS analysis, were specifically confirmed by low-throughput experimental studies [35]. In the present work, for the first time, we identified LPAP phosphorylation sites using a complex of different but complementary methods, including site-directed mutagenesis, phospho-specific antibodies, and specific immunoprecipitation/MS strategies. Our results not only confirm the Ser-99, Ser-153, and Ser-163 phosphosites reported previously, but also provide evidence for a new phosphorylation site at Ser-172.

LPAP Ser-99 phosphorylation has been reported by many groups [15–19,22–24] due to the location of Ser-99 site in a MS-friendly tryptic peptide. A casein kinase I (CK1) substrate motif, [E/D]XX[S/T], is located close to Ser-99 [36], and an acidic cluster, DLERQEDEQD, downstream from Ser-99, can potentiate the activity of CK1 [36]. Furthermore, Ser-99 is phosphorylated both in human LPAP [16] and in murine PTPRCAP [17,37].

The most conserved region of the LPAP cytoplasmic domain is the C-terminus (aa 169–206) where Ser-172 is located. We demonstrated that in human LPAP, Ser-172 is a phosphorylation site, and these data are supported by a recent study [37], which detected the phosphorylation of Ser-163 located in a homologous area of the murine protein. However, the kinase responsible for the phosphorylation of Ser-172 is unclear, although we hypothesize that it could be casein kinase II (CK2), which is able to phosphorylate the SXE motif [38]. CK2 has a very broad specificity, acting on more than 300 different protein substrates [36]; however, the SXE motif found in LPAP is not optimal for this kinase.

Ser-153 in human LPAP is homologous to the Thr-144 site in murine PTPRCAP. Both sites are located in the acid motif D[S/T]D[T/S][E/D]G, and though high levels of phosphorylation are present in the human protein, phosphorylation of murine PTPRCAP Thr-144 has not been identified. Presumably, this is due to the fact that after trypsinolysis, Thr-144 is placed in a 51-aa peptide with a molecular weight of 4750 Da, which is not easily analyzed by MS. Ser-153 is positioned within a typical CK2 recognition motif, SXXE/D; however, in LPAP, we observe the more specific motif [pS][D/E]X[D/E]X[D/E] [39].

Unlike Ser-99 and Ser-172, which are phosphorylated dynamically, Ser-153 is believed to be stably phosphorylated. Ser-153 is positioned in the acidic Lck binding domain of LPAP [4,37]. The high level of phosphorylation within the Lck binding domain may be assumed to control the association of LPAP with Lck. However, we previously reported that the level of LPAP phosphorylation is not dependent on Lck [27].

**Fig 9:**
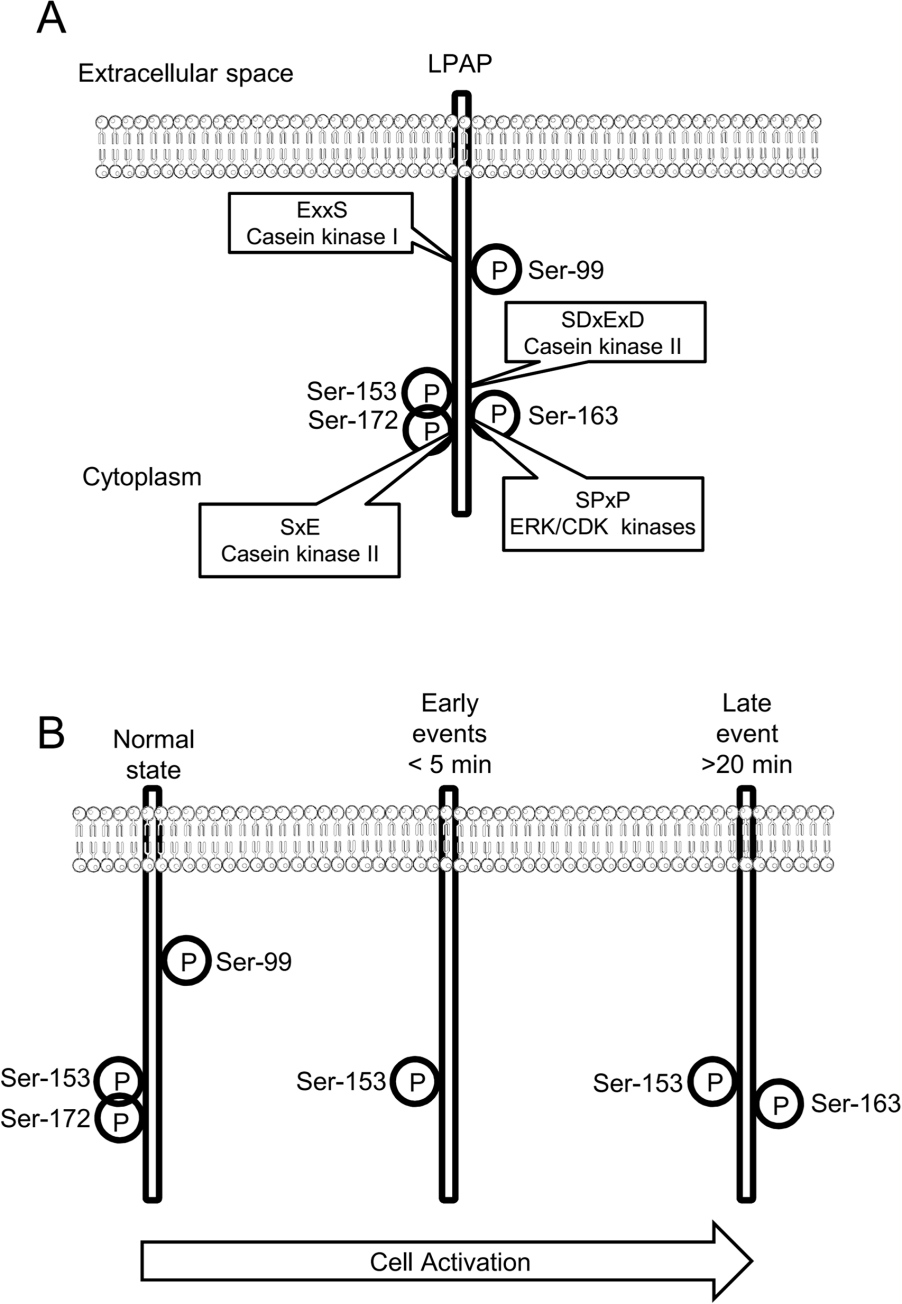
Schematic representation of LPAP phosphorylation sites and motifs. (A) Scheme indicates the positions of identified LPAP phosphorylation sites, motifs in which they are located and kinases which may be responsible for LPAP phosphorylation. (B) In resting normal lymphocytes LPAP is present as a mixture of mono-, di-, and three-phosphorylated proteoforms with phosphogroups located at Ser-99, Ser-153, and Ser-172. Soon after PMA activation pSer-99 and pSer-172 are dephosphorylated by an unknown phosphatase. However, by 20 min of activation Ser-163 is phosphorylated presumably by ERK/CDK kinases. P, phosphorylation.

Ser-163 phosphorylation has been achieved by stimulating CD3/CD28 receptors [13], and is part of the SPxP motif, which is recognized by proline-directed kinases such as cyclin-dependent kinases (CDKs) and mitogen-activated protein kinases (MAPK) [39]. A summary of LPAP phosphorylation sites and kinase motifs is presented in Fig 9A.

The cytoplasmic tail of LPAP contains three tyrosine residues, two of which, Tyr-64 and Tyr-115, are predicted to be phosphorylated with a high score by NetPhos. Tyr-115 is part of a consensus sequence, YxxV/L/I, for phosphorylation by Src family protein tyrosine kinase (PTK) [40], and a small portion of Tyr is indeed phosphorylated [29]. Nevertheless, we failed to detect any phosphorylation at Tyr-115 by site-directed mutagenesis, anti-pTyr Ab staining, or MS analysis.

In contrast to protein kinases, Ser-Thr phosphatases from the most abundant group of PPP phosphatases do not have well-defined motifs and the prediction of their potential substrates is not straight-forward [41]. There are several degenerate motifs for Ser-Thr phosphatases reported in the literature (in comparison to more than a hundred of motifs for kinases), but none of them is found in LPAP [42]. Thus, the nature of LPAP phosphatases is still under question.

Conventionally, proteomic data concerns individual phosphorylation sites and does not reflect the presence of site combinations, but this information can be deduced from analysis of particular phosphoforms. Using 2D-PAGE and Phos-tag-PAGE, we resolved and quantitatively characterized at least six LPAP phosphoforms, providing the first quantitative measurement of LPAP phosphorylation at individual sites. Furthermore, we found that Ser-153 was phosphorylated in most LPAP molecules, whereas in resting CEM cells, Ser-99 and Ser-172 were phosphorylated in 35% and 15% of LPAP molecules, respectively.

It is important to determine whether LPAP phosphorylation characterized for CEM cells is valid for primary lymphocytes. We found that patterns of LPAP phosphorylation detected by SDS-PAGE in PBMCs resembled those in CEM cells. Therefore, we considered the phosphorylation sites identified in LPAP from CEM cells appropriate for LPAP from PBMCs. However, there is some uncertainty about phosphorylation of Ser-172 in lymphocytes. Thus, we could not detect a downward shifted band at 28 kDa in purified CD4^+^ and CD8^+^ subsets. In line with this phospho-specific anti-p172 Abs gave poor reaction with LPAP from PBMC, possibly due to low stoichiometry of pSer-172 in these cells.

In order to understand how proximal or distal LPAP phosphorylation takes place in a signaling pathway, we compared the kinetics of LPAP phosphorylation changes after PMA stimulation with the time courses of other PMA-induced processes. PMA is a direct PKC activator that promotes rapid (<5 min) redistribution of PKC from the cytosolic to the membrane compartment [43]. PMA stimulation also leads to activation of the Ras signaling pathway. The PMA-triggered phosphorylation of p42/44 MAPK occurs by 2 min, and the phosphorylation of p38 MAPK and p90Rsk increases more slowly and peaks by 15–30 min [44–46]. Eventually, PMA activates the transcription factors nuclear factor (NF)-kB, nuclear factor of activated T cells (NFAT), and others. However, this is a delayed process that requires several hours [47–49]. Compared to the processes described above, the dynamic of pSer-99 and pSer-172 dephosphorylation is rapid (<5 min), and therefore, related to early events associated with PMA-induced cell stimulation. This supports the idea that LPAP dephosphorylation at pSer-172 is proximal to PMA stimulation and is not the effect of secondary processes. Similarly, Mayya et al. [16] demonstrated that Ser-99 is dephosphorylated after activation of lymphocytes through the TCR. In contrast, the phosphorylation kinetics of Ser-163 were significantly delayed, suggesting that they are mediated by a kinase working at the late signaling steps, presumably by extracellular signal-regulated kinase (ERK), a proline-directed kinase (Fig 9).

We suggest that the phosphorylation of LPAP is involved in signal transduction in lymphocytes for the following reasons. First, LPAP is localized to the cell membrane. Second, LPAP is tightly associated with the following molecules involved in the regulation of lymphocyte signaling: protein tyrosine phosphatase CD45 [29], co-receptor CD4 [6], and kinase Lck [4]. Third, the kinetics of LPAP phosphorylation are consistent with those observed for other major signaling molecules. Taken together, these data make it possible to hypothesize that LPAP plays a role in cell signaling.

In summary, our study demonstrated that LPAP in lymphocytes exists in several phosphoforms, which we detected through site-directed mutagenesis, phospho-specific antibodies, and MS analysis. We have shown that phospho-modification of LPAP is altered dynamically and site-specifically upon cell stimulation with the PKC activator, PMA. We believe that the changes in the phosphorylation status of LPAP are important for TCR activation and signal transduction in primary human lymphocytes, and its comprehensive analysis may ultimately uncover a true function of this small enigmatic molecule.

## Supporting information

S1 Fig Supplementary images to Fig 1. (PDF)

S2 Fig Supplementary images to Fig 3. (PDF)

S3 Fig Supplementary images to Fig 4. (PDF)

S4 Fig Supplementary images to Fig 5. (PDF)

S5 Fig Supplementary images to Fig 6. (PDF)

S6 Fig Supplementary images to Fig 7. (PDF)

S7 Fig Supplementary images to Fig 8. (PDF)
