# Supplementary figures and images for: Constitutive and activation-dependent phosphorylation of lymphocyte phosphatase-associated phosphoprotein (LPAP)
Source: PLoS One. 2017 Aug 21;12(8):e0182468. doi: 10.1371/journal.pone.0182468 (PMC5565103; doi:10.1371/journal.pone.0182468)

A

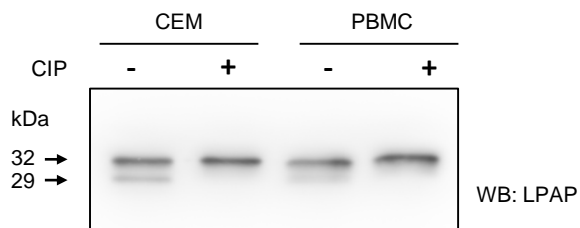

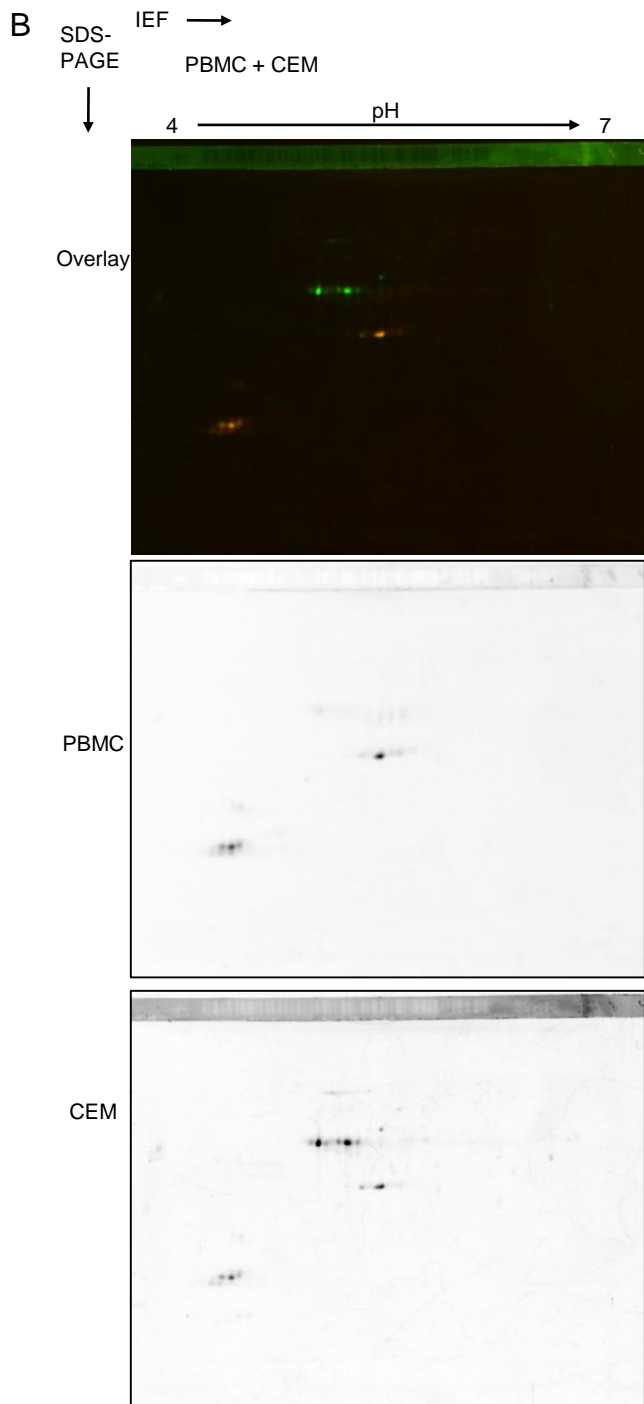

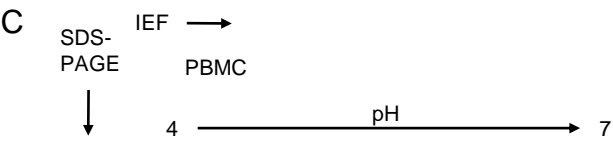

Overlay

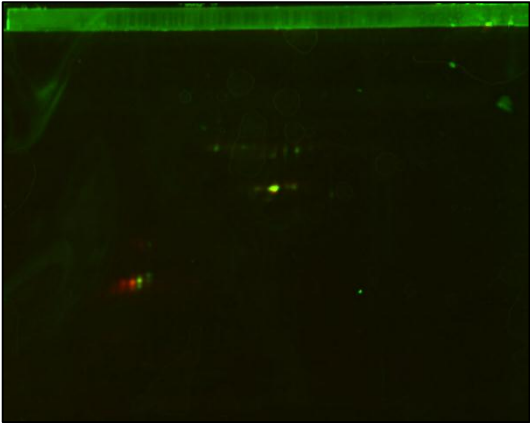

untreated

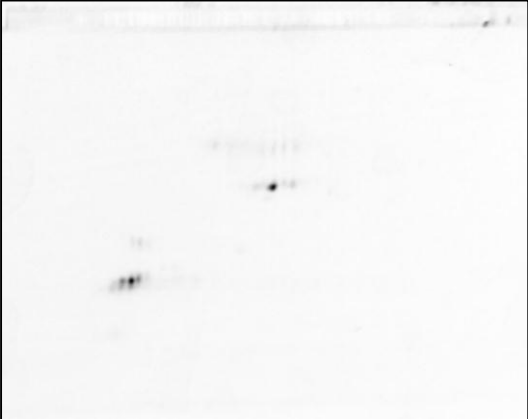

+CIP

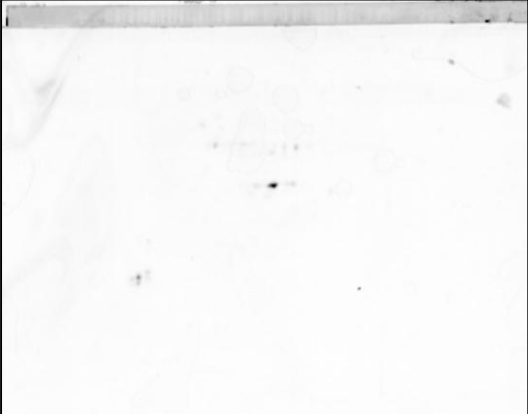

Supplement: S1 Fig — (PDF) [file pone.0182468.s001.pdf]

B

CEM CEM98 S99A S153A S163A S168A S172A

32 kDa→

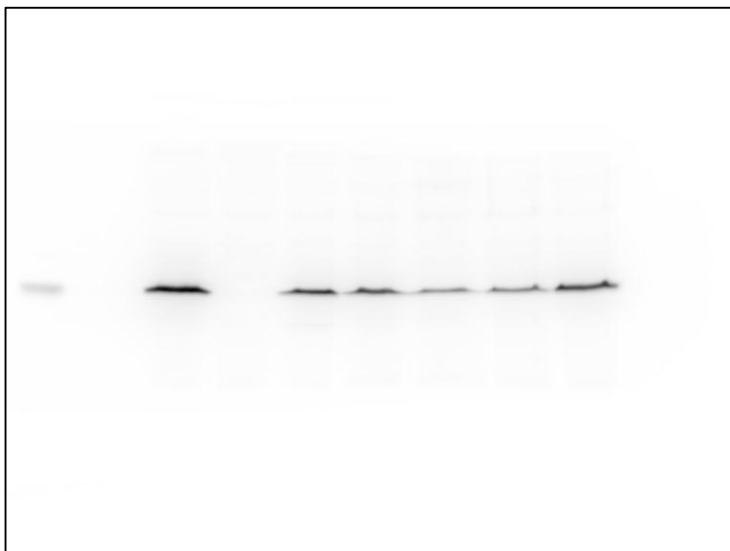

WB:LPAP

50 kDa→

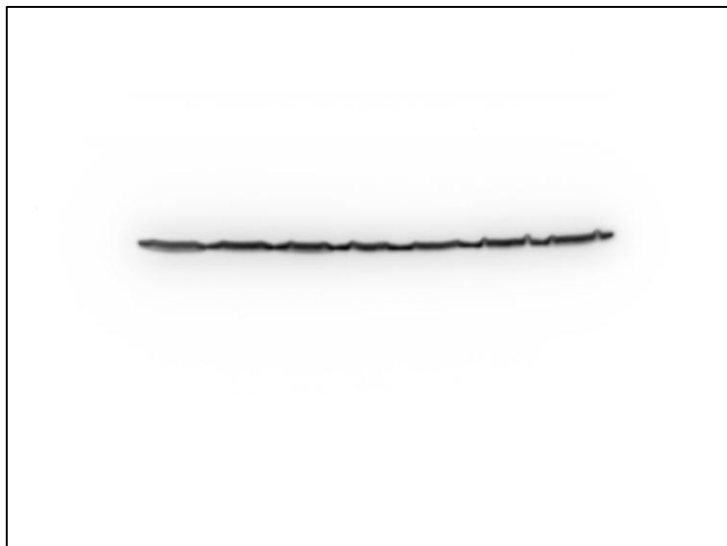

WB:tubulin

D

SDS-  
PAGE

IEF  
4

pH

7

Overlay

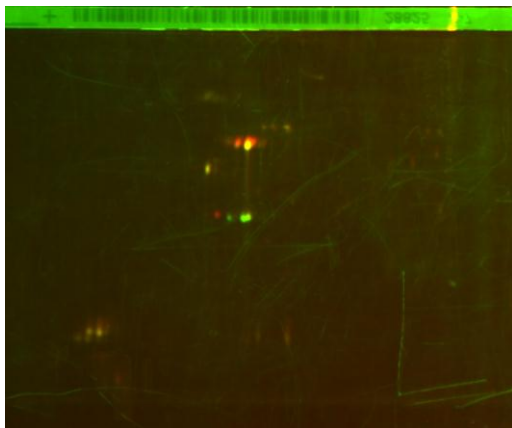

CEM

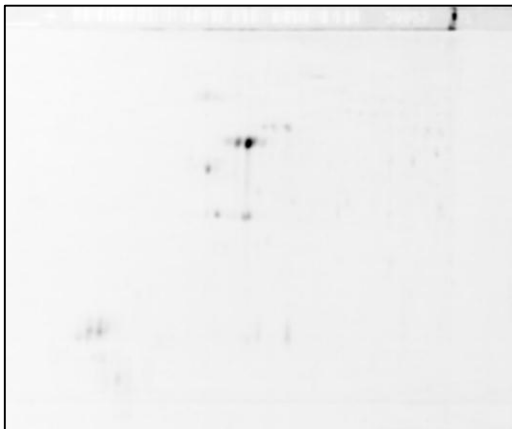

CEM98  
LPAP-wt

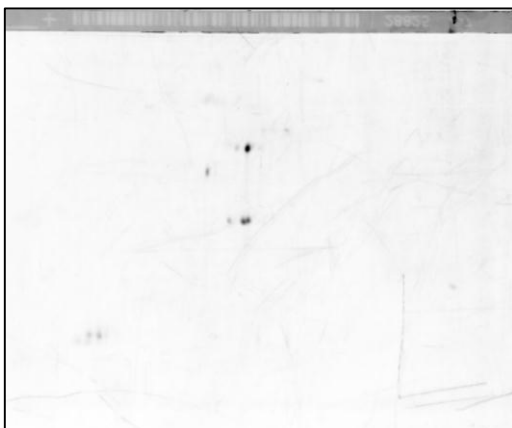

Supplement: S2 Fig — (PDF) [file pone.0182468.s002.pdf]

A

|     | CEM |   | S163A |   | S168A |   | S172A |   | S163A,<br>S168A,<br>S172A |   |
|-----|-----|---|-------|---|-------|---|-------|---|---------------------------|---|
| CIP | -   | + | -     | + | -     | + | -     | + | -                         | + |

32 kDa→

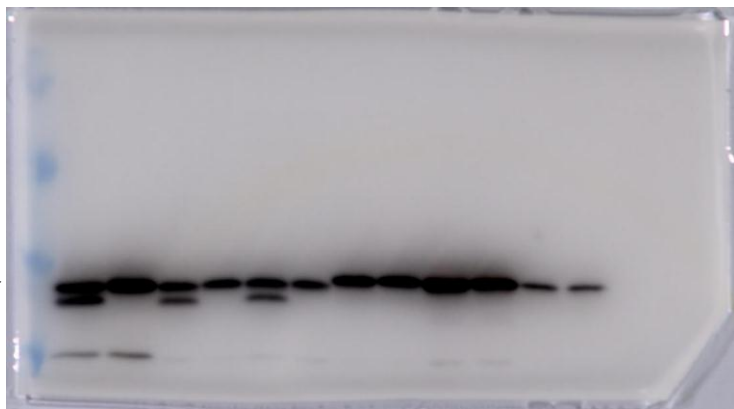

B

SDS-  
PAGE

IEF →

4 → pH → 7

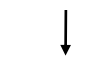

Overlay

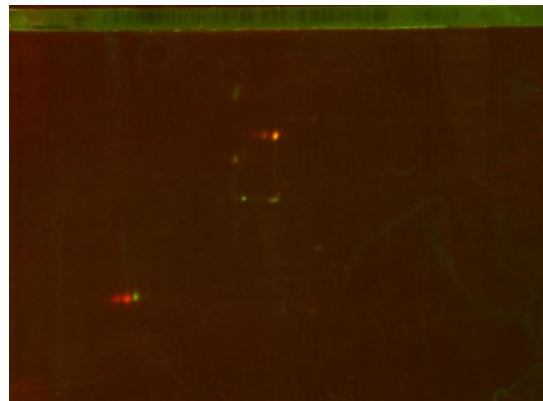

-CIP

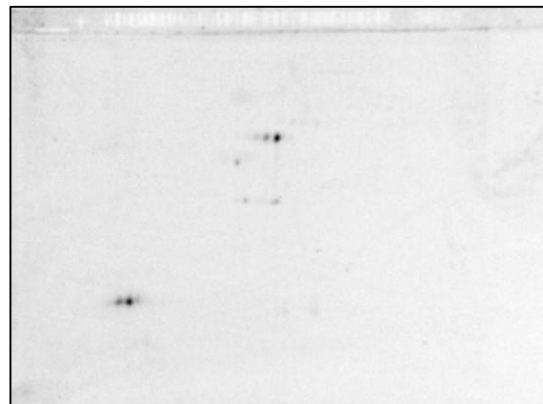

+CIP

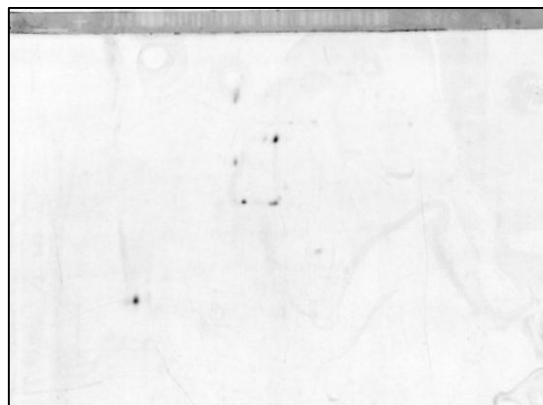

Supplement: S3 Fig — (PDF) [file pone.0182468.s003.pdf]

A

SDS-  
PAGE

IEF

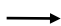

4 pH 7

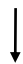

Overlay

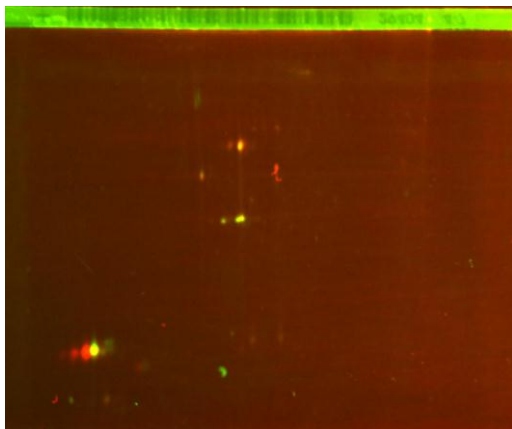

-CIP

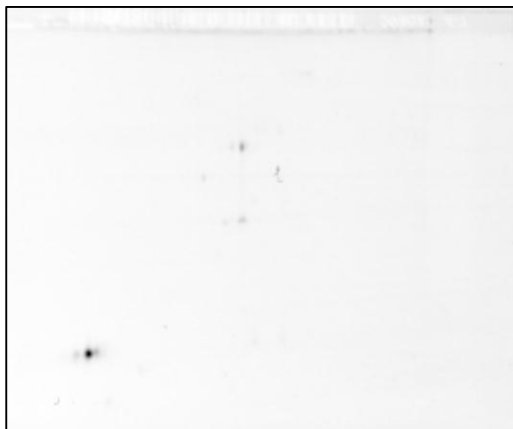

+CIP

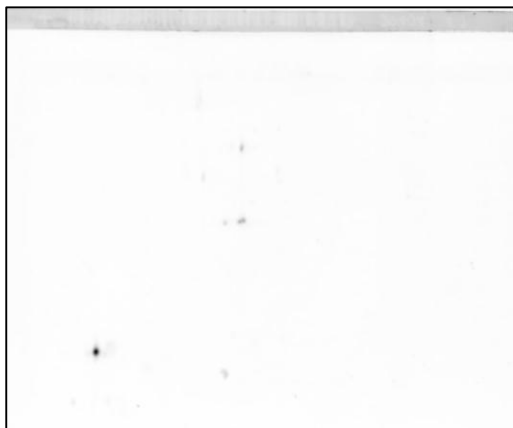

Supplement: S5 Fig — (PDF) [file pone.0182468.s005.pdf]

A

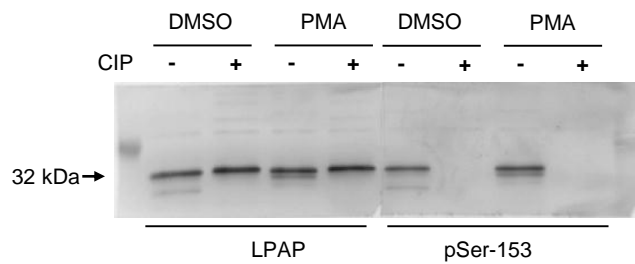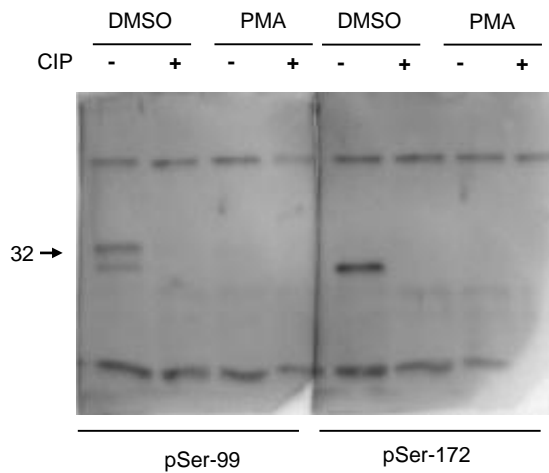

B

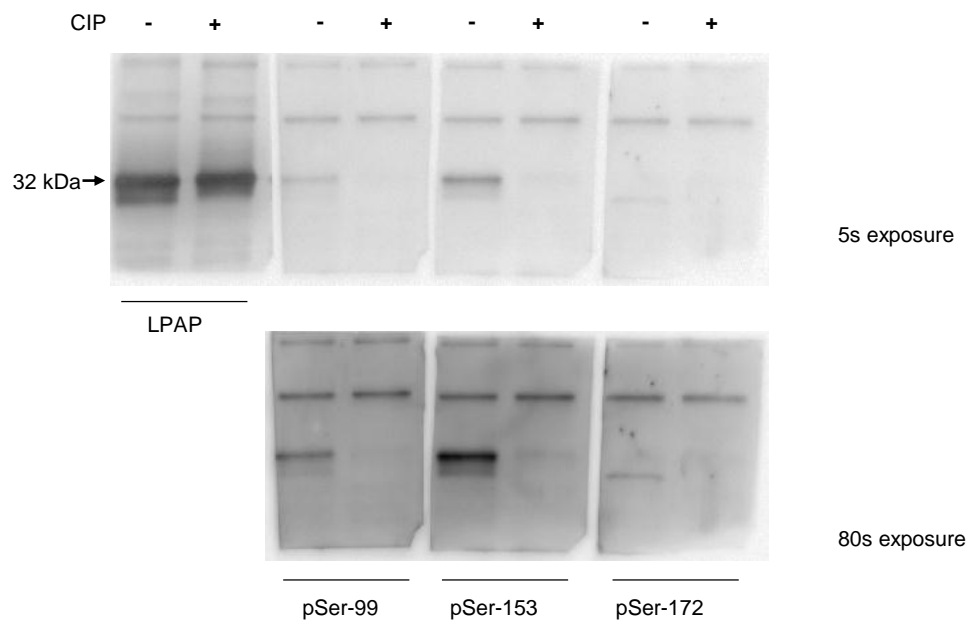

C

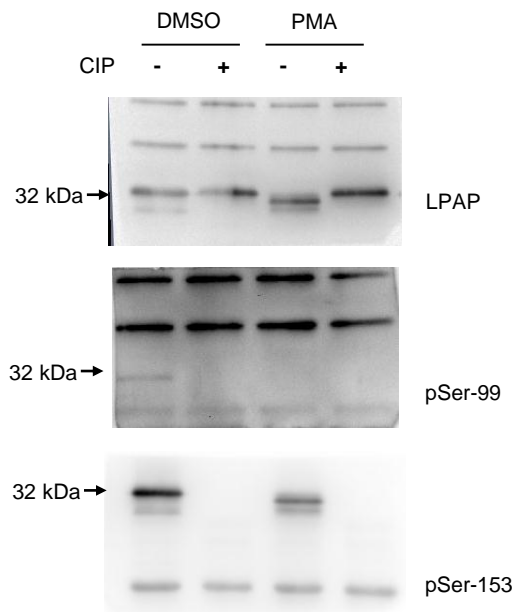

Supplement: S6 Fig — (PDF) [file pone.0182468.s006.pdf]

A

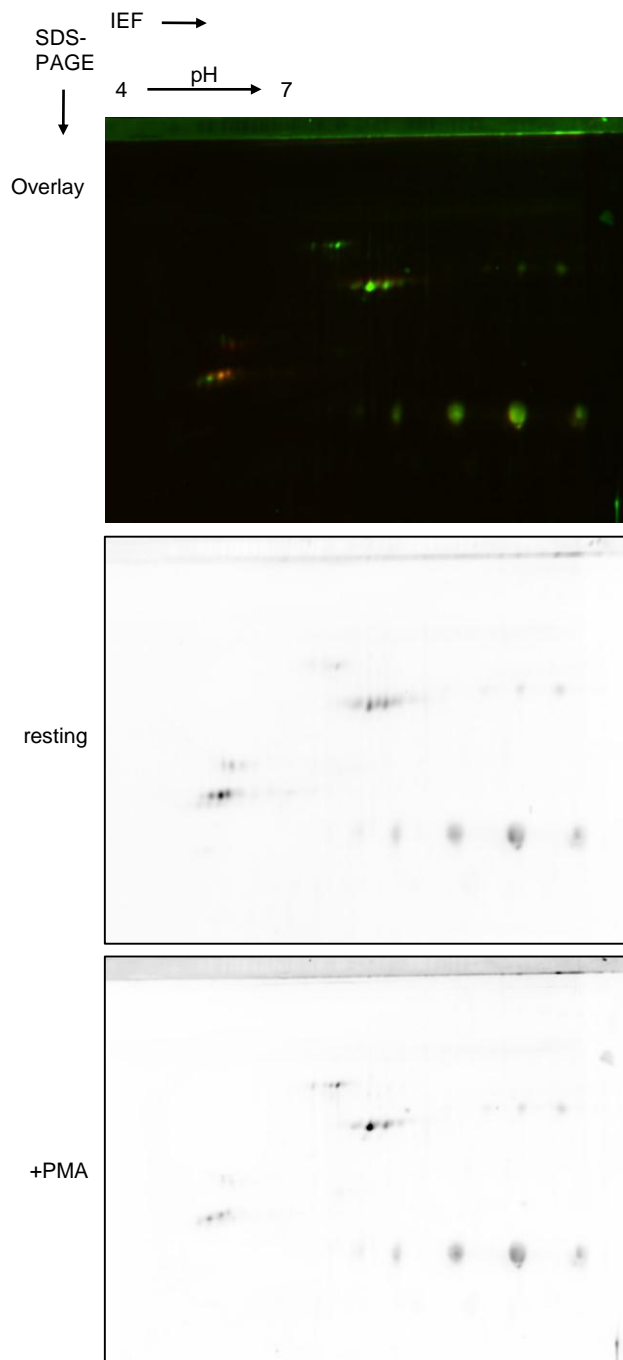

Supplement: S7 Fig — (PDF) [file pone.0182468.s007.pdf]
