# Supplementary material for: Constitutive and activation-dependent phosphorylation of lymphocyte phosphatase-associated phosphoprotein (LPAP)
Source: PLoS One. 2017 Aug 21;12(8):e0182468. doi: 10.1371/journal.pone.0182468 (PMC5565103; doi:10.1371/journal.pone.0182468)
Supplement: S4 Fig — (PDF) [file pone.0182468.s004.pdf]

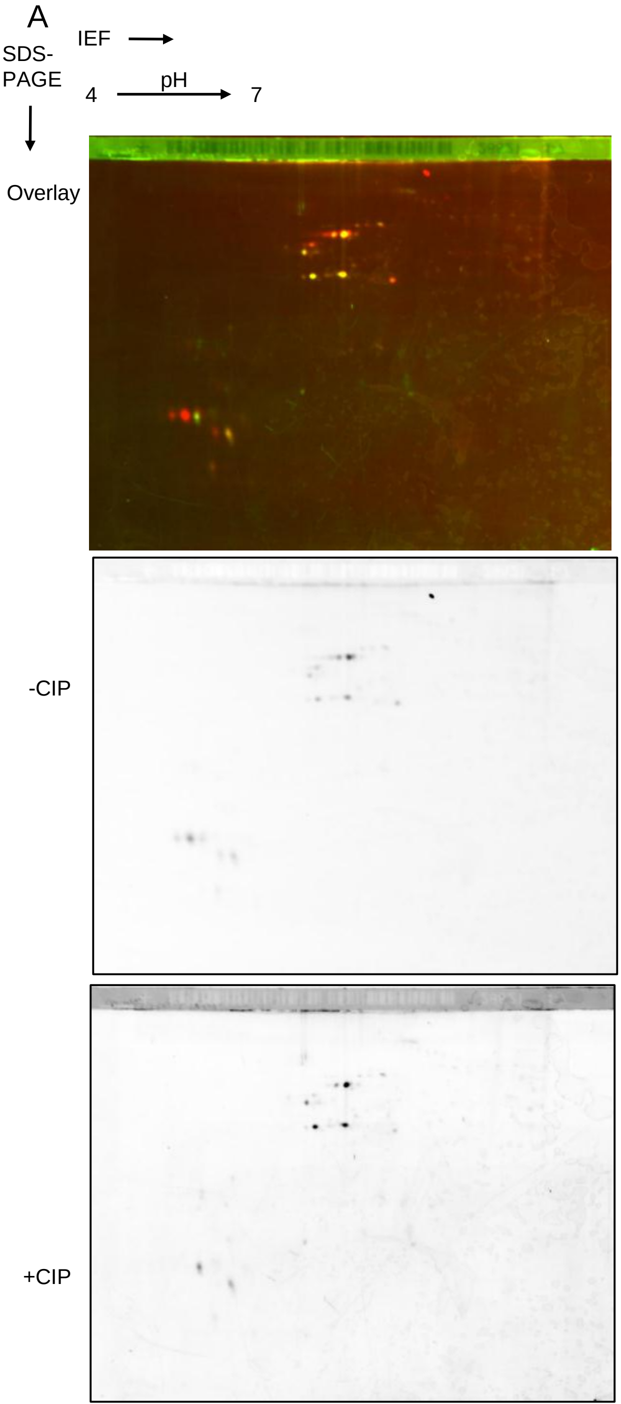
A
IEF
SDS-
PAGE
4
pH
7
Overlay
-CIP
+CIP

B

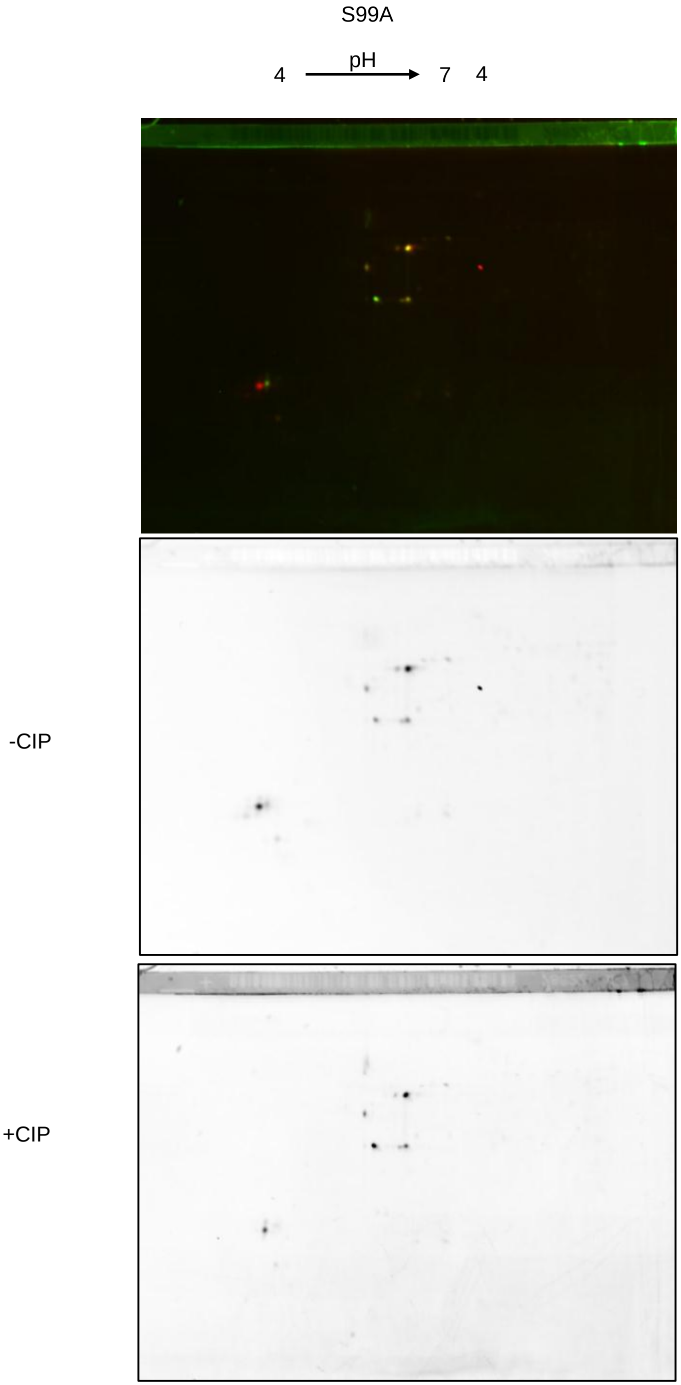

B

S153A

pH

4 → 7

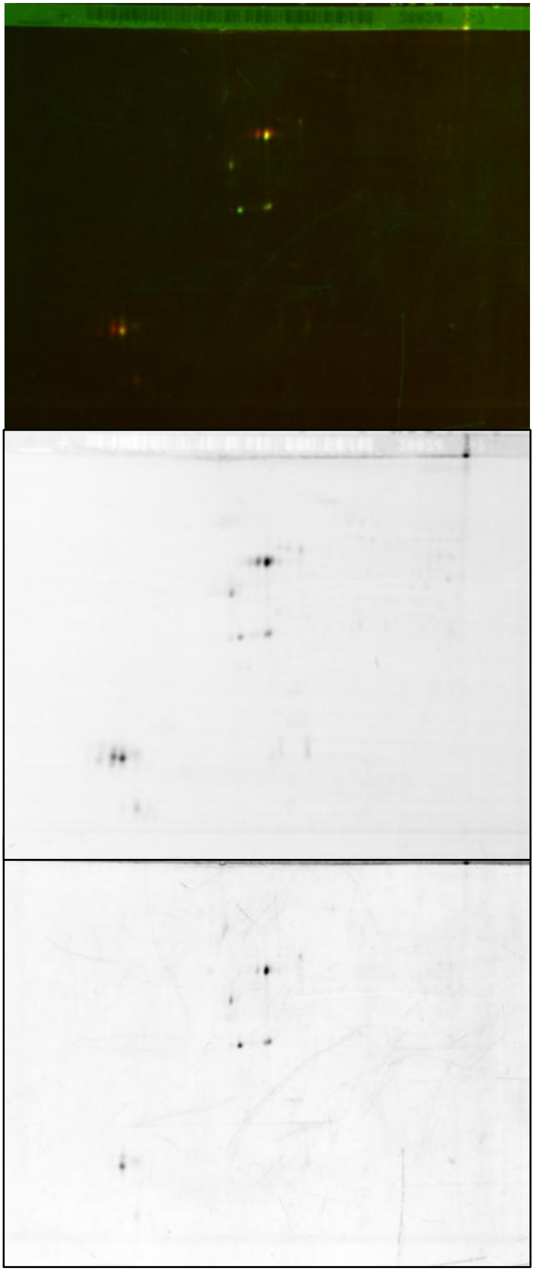

-CIP

+CIP

B

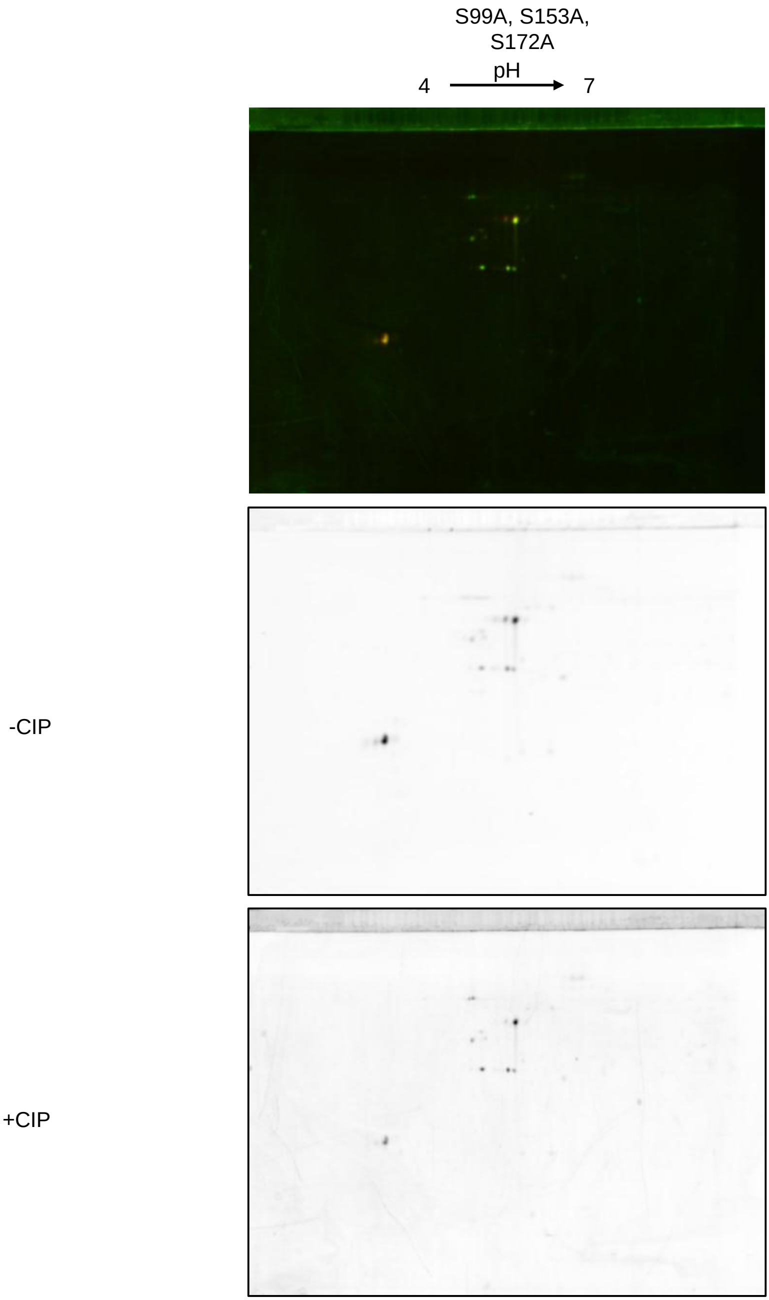

B

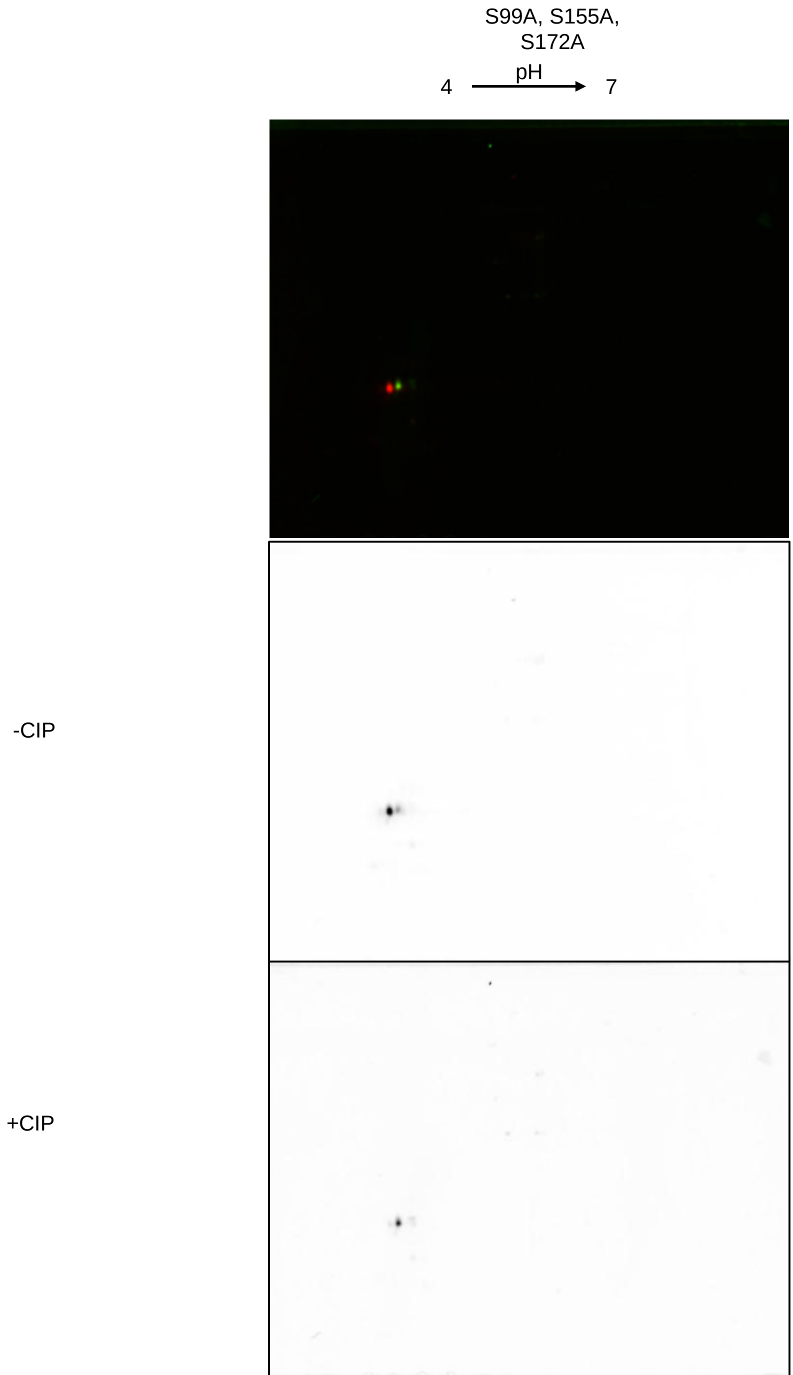

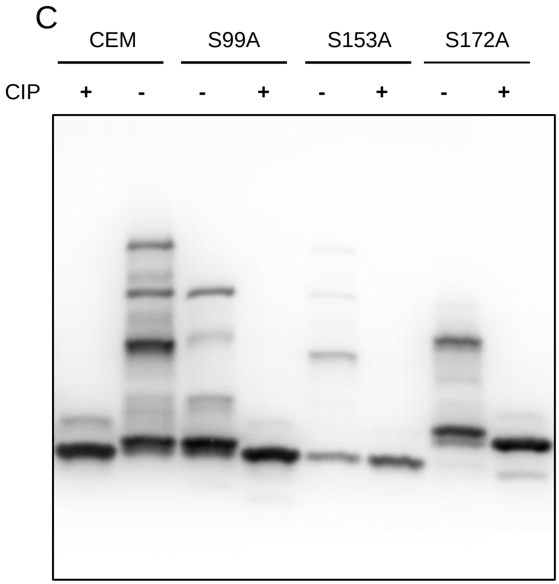

Phos-tag SDS-PAGE
